# Alginate Oligosaccharide and Gut Microbiota: Exploring the Key to Health

**DOI:** 10.3390/nu17121977

**Published:** 2025-06-11

**Authors:** Meiling Song, Lin Chen, Chen Dong, Minghui Tang, Yuan Wei, Depeng Lv, Quancai Li, Zhen Chen

**Affiliations:** 1 School of Pharmacy, Jiangsu University, Zhenjiang 212013, China; songmeiling@stmail.ujs.edu.cn (M.S.); chenlin@stmail.ujs.edu.cn (L.C.); dongchen@stmail.ujs.edu.cn (C.D.); ywei@ujs.edu.cn (Y.W.); 2 Department of Diagnostic Imaging, Faculty of Medicine and Graduate School of Medicine, Hokkaido University, Sapporo 060-8638, Japan; 3 Marine Biomedical Research Institute of Qingdao, Qingdao 266071, China; lvdepeng@ouc.edu.cn; 4 Key Laboratory of Marine Drugs, Ministry of Education, Shandong Provincial Key Laboratory of Glycoscience and Glycoengineering, School of Medicine and Pharmacy, Ocean University of China, Qingdao 266003, China

**Keywords:** alginate, β-D-mannuronic acid, α-L-guluronic acid, oligosaccharide, gut microbiota, prebiotics, intestinal health, short-chain fatty acids

## Abstract

Alginate oligosaccharide (AOS), a degradation product of alginate derived from marine brown algae, has attracted significant attention due to its potent ability to modulate gut microbiota and enhance human health. This review aims to systematically introduce current evidence on the interactions between AOS and gut microbial communities, focusing on how AOS improves health through regulating gut microbiota. Initially, the structural factors of AOS that influence their functions are highlighted, including molecular weight, monomer composition, terminal structure, and chemical modifications. Importantly, AOS primarily exerts beneficial effects by adjusting gut microbiota community and outputs, which include the promotion of probiotics, the inhibition of pathogens, the balance of microbiota composition, and the increase of short-chain fatty acid production. Moreover, the discovered mechanisms underlying AOS-mediated health promotion via microbiota modulation are detailed comprehensively, specifically emphasizing intestinal barrier maintenance, antioxidation, dual-regulation of immune and inflammatory responses, pathogenic infection inhibition, metabolic improvement, uric acid excretion promotion, anti-tumor effects, and anti-skin aging. Such beneficial effects make AOS valuable in keeping healthy, preventing disorders, and intervening in diseases. Despite these findings and research progress, there are yet limitations in studying AOS–gut microbiota interactions, such as precise microbiota-targeted structural optimization, personalized nutritional interventions based on microbial characteristics, and broadening the horizon of microbiota-derived metabolic metabolomic profiles. In conclusion, advancing our understanding of the gut microbiota-centered mechanisms of AOS would probably facilitate novel nutritional strategy development for health promotion.

## 1. Introduction

The human gut microbiota, a complex and dynamic community of microorganisms residing in the gastrointestinal tract and usually called gut microbiota, gut microbiome, or gut flora, plays crucial roles in human health. It harbors a vast array of genes that complement the host’s genome and contribute to various physiological processes, and thus is often referred to as the “second genome” [1,2]. This intricate microbial ecosystem plays essential roles in nutrient metabolism, immune system modulation, and maintenance of gut barrier integrity. For instance, the gut microbiota is involved in the fermentation of indigestible dietary fibers, producing short-chain fatty acids (SCFAs) as the energy sources for colonocytes, and participating in the function of the immune system, to protect the host against pathogens and maintain immune homeostasis [3,4]. The imbalance of gut microbiota, also known as dysbiosis, has been increasingly recognized as a critical factor in the development of various diseases, such as inflammatory bowel diseases (IBDs) (e.g., Crohn’s disease and ulcerative colitis) [5], metabolic disorders (e.g., diabetes, obesity, and fatty liver disease) [6,7,8,9], and neurodegenerative diseases (e.g., Alzheimer’s and Parkinson’s disease) [10].

**Figure 1:**
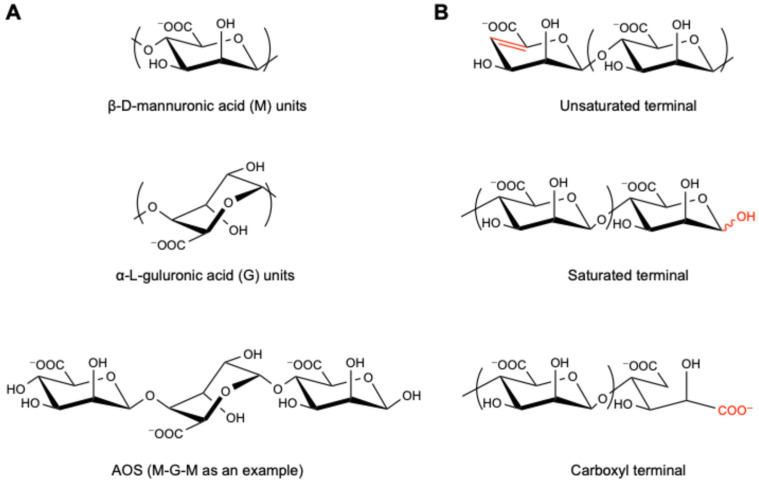
The structure of AOS. (**A**) β-d-mannuronic acid (M) and α-l-guluronic acid (G) units, along with one of their oligomers, “M-G-M”, as an example; (**B**) terminal structures of unsaturated, saturated, and carboxyl AOS. The red color highlights the difference of terminal structures.

Research has long focused on the role of nutritional ingredients in regulating gut microbiota. In recent years, attention has been drawn to the potential of marine algae, applied in not only agriculture and materials but also foods and medicines [11,12,13]. Substantial progress has especially been made in the exploration of alginate, a kind of linear acidic polysaccharide [14] primarily derived from edible brown seaweeds such as kombu (*Saccharina japonica*), wakame (*Undaria pinnatifida*), and hijiki (*Sargassum fusiforme*) [15,16,17]. Serving as the degradation products of alginate (Figure 1), alginate oligosaccharide (AOS) has been recognized to possess various health-beneficial functions, including immune-enhancing, anti-inflammatory, antioxidant, and even anticancer effects [18,19,20,21]. More importantly, researchers have revealed that AOS influences gut microbiota by regulating their growth and activities, consequently offering promising therapeutic potential in addressing gut-related diseases and improving the health of the hosts [21,22]. Nowadays, the global market value of AOS, together with alginate and other derivatives, has reached approximately 500 million US dollars [16]. Among these, the new drug “GV-971” (sodium oligomannate capsules) was approved by the National Medical Products Administration (NMPA) of China in 2019.

The bioavailability of AOS is relatively low. After oral administration, only a small amount of AOS is absorbed, among which the majority exists in the circulating body fluid in the free form, and the trace distributes in tissues (such as brain and liver) and hemocytes. Moreover, the absorbed AOS hardly undergoes metabolism and excretes in its original form. Nevertheless, the pharmacokinetic parameters are influenced by administration methods, dosages, animal species, individual variations, and structural compositions of AOS. In comparison, most AOS is fermented by the gut microbiota [23,24]. While numerous reports have focused on the preparation and characterization of AOS, some researchers have been investigating its interactions with gut microbiota. It is of increasing interest to discuss how AOS improves body health through its actions on the gut microbiota.

Based on these clues, this review aims to explore the role of AOS in modulating gut microbiota and the consequent implications for health. The relationships between the AOS structure and gut microbiota will be summarized. Moreover, the effects of AOS on potential therapeutic applications will be intensively discussed.

## 2. The Impact of AOS Structure on Biological Activities

### 2.1. Molecular Weight or Degree of Polymerization

The molecular weight of AOS is related to the degree of polymerization (DP), which refers to the number of β-d-mannuronic acid (M) and/or α-l-guluronic acid (G) monomer units (Figure 1A). Studies have shown that the beneficial effects of AOS vary along with their DP. For instance, Peng et al. compared different AOS fractions for their safety and impact in soybean seeds and found that AOS with a larger molecular weight (i.e., higher DP) showed higher glyceollin-inducing activities [25]. In parallel, Chen et al. reported that only AOS5 (i.e., DP = 5) showed anti-tumor effects on human bone cancer cell MG-63 in vitro among the prepared fractions (DP = 2, 3, 4, and 5; with molecular weight of 414, 612, 810, and 1009 Da, respectively) [26]. Iwamoto et al. revealed that AOS fractions with a DP of 7 or 8 showed better potential as immunotherapeutic agents in RAW264.7 cells [27], including the induction of tumor necrosis factor (TNF-α), interleukin-1α (IL-1α), interleukin-1β (IL-1β), and interleukin-6 (IL-6). Another study by Yin et al. focused on the ankle swelling inhibition activity of the AOS fractions with lower molecular weight (DP from 2 to 4), in which AOS2 and AOS3 exhibited much greater analgesia in the gouty arthritis model mice compared with AOS4 [20]. Although these studies indicated a certain correlation between DP and the bioactivity of AOS, a definite pattern has not yet been discovered.

### 2.2. Composition of M and G, and Their Ratio

The bioactivities of AOS are significantly influenced by its constituents of M and G, as well as the M/G ratio. In alginate, the M-block, characterized by a flexible linear structure [28,29], exhibits more remarkable advantages in the aspects of regulating metabolism, preserving the nervous system, and improving inflammation than the G-block [30]. Oligomannuronate AOS (MAOS) can ameliorate tauopathies by enhancing the aggregation of the Tau-K18 oligomer and lowering the level of phosphorylated Tau protein, as well as promoting autophagy [31]. In addition, it is recognized to regulate the gut microbiota structure to increase the production of 1,2-dioleoyl-sn-glycero-3-phosphocholine, which leads to the improvement of experimental colitis and secondary neurological dysfunction via regulating the gut–brain axis and restraining the activation of the TLR4/MyD88/NF-κB signal pathway [32]. Moreover, Lu et al. investigated the structure–function relationship of AOS by comparing MAOS, oligoguluronate AOS (GAOS), and heterogeneous AOS (HAOS) and found that only MAOS significantly relieved colon shortening, reduced disease activity index (DAI) and histology score, and decreased the levels of IL-6 and IL-8, effectively preventing colon inflammation, reversing gut barrier function impairment, and regulating gut microbiota composition, therefore leading to alleviation of colitis symptoms in a dextran sodium sulfate (DSS)-induced mouse model [33]. Furthermore, Gao et al. claimed that MAOS mediated gut–skin axis (butyrate–HIF-1α) homeostasis, improving skin aging, altering gut microbiota, and maintaining skin mitochondrial function and energy supply in mice, of which the effects were much more significant than GAOS and HAOS [34].

On the other hand, the G units, contributing to a more rigid, folded conformation, alter the physicochemical properties of AOS [35], potentially affecting their interactions with biological targets. GAOS exhibits a better effect in enhancing immunity and protecting against pathogens [30]. Fang et al. demonstrated that GAOS could be absorbed by macrophages, leading to the activation of TLR4/Akt/NF-κB, TLR4/Akt/mTOR, and MAPK signaling pathways, and eventually enhancing immunity [36]. Notably, a new alginate oligosaccharide drug, OligoG (G-block fragment from AOS), has been shown to prevent lung infections by disrupting *Pseudomonas aeruginosa* biofilm in a dose-dependent manner [37]. Also, it has been verified to bind to the bacterial surface, thereby inducing microbial aggregation and inhibiting motility [38]. Furthermore, van Koningsbruggen-Rietschel et al. reported inhaling dry powder of OligoG as a new, safe, and effective therapy for treating chronic gram-negative infections in cystic fibrosis patients [39].

In terms of HAOS, as a mixture of both M and G units, it is recognized for its ability to prevent oxidative stress and apoptosis. HAOS could inhibit H_2_O_2_-induced oxidative stress (as endoplasmic reticulum (ER) and mitochondrial-dependent apoptotic cell death) to protect PC12 cells [40], reducing the intracellular reactive oxygen species (ROS) levels through activating the nuclear factor erythroid 2-related factor 2 (Nrf2) signaling pathway in the GES-1 gastric cell line [41], upregulating ROS scavenging activities and attenuating the caspase-mediated apoptosis pathway to enhance their antioxidant and antiapoptotic activities in H_2_O_2_-stressed human umbilical vein endothelial cells [42]. Moreover, in the doxorubicin-induced oxidative stress model, HAOS (M/G ratio = 0.4) suppressed the expressions of gp91 (phox) and 4-hydroxynonenal (4-HNE), downregulated the expression of Caspase-12, C/EBP homologous protein, and Bax, and upregulated the expression of anti-apoptotic protein Bcl-2 to prevent acute doxorubicin cardiotoxicity [43].

### 2.3. Terminal Structure

Generally, unsaturated AOS (UAOS) is derived through enzymatic degradation, which is highly specific and with mild reaction conditions, and thus more accepted and recommended, while saturated AOS (SAOS) is obtained by acid and physical degradation methods [44], which enables large-scale production (Figure 1B). UAOS shows a better performance in regulating immunity and improving lipid metabolism and could reduce body weight, serum lipids, including triacylglycerol (TG), total cholesterol (TC), and free fatty acids (FFAs), liver weight, liver TG and TC, serum alanine aminotransferase (ALT), and aspartate aminotransferase (AST) levels, adipose mass, ROS formation, and accumulation induced in mice with a high-fat diet [45]. Also, Iwamoto et al. revealed that UAOS induced TNF-α secretion from RAW264.7 cells in a structure-dependent manner, while such activity of SAOS prepared by chemical degradation was fairly low or showed only trace levels [27]. Furthermore, the unsaturated MAOS has been uncovered to play an important role in improving Alzheimer’s disease, which depended on suppressing the aggregation and expression of amyloid-β (Aβ)_1–42_ oligomer, downregulating the content of amyloid precursor protein (APP) and BACE1, and promoting the fusion of autophagosomes and lysosomes via activating the mTOR signaling pathway [46]. In addition, according to Xu et al., the GAOS with an unsaturated terminal structure could activate the NF-κB and MAPK signaling pathways, resulting in significant induction of nitric oxide (NO), ROS, and TNF-α production in RAW264.7 macrophage activation [47].

Besides UAOS and SAOS, the AOS prepared through oxidative degradation (e.g., Fenton reaction) usually possesses the terminal dicarboxyl structure (Figure 1B) [48]. For instance, Zhou et al. compared the anti-inflammatory activity of GAOS prepared by different methods and found that those prepared through oxidative degradation exhibited a more significant effect in lipopolysaccharide-activated murine macrophage RAW264.7 cells than those prepared by enzymatic degradation or acid hydrolysis [49], suggesting that the terminal structures of AOS crucially affect their bioactivities.

Beyond the bioactivities varying from its diverse natural structures, AOS can also be further modified, such as by sulfation, selenylation, and even vanadylation, to generate AOS-based derivatives, which often result in enhanced or even new activities [48].

## 3. Multiple Influences of AOS on Gut Microbiota

**Figure 2:**
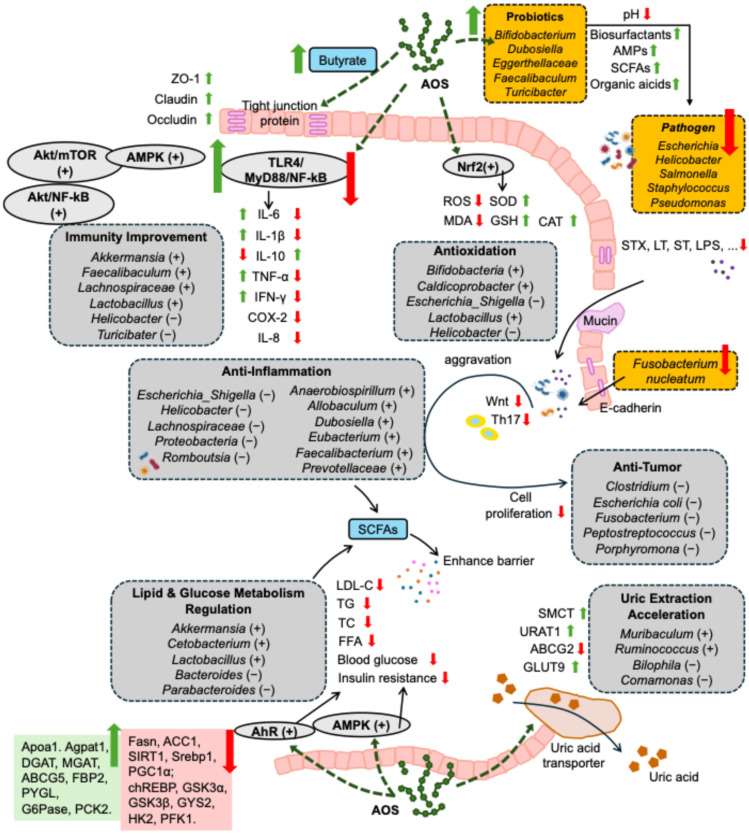
Mechanism of AOS in promoting health through gut microbiota, including maintenance of intestinal barrier integrity (involving upregulating the expression of intestinal tight junction and increasing the thickness of intestinal mucus layer), antioxidation (involving regulating the oxidative and antioxidative system homeostasis), dual regulation of inflammation and immune responses, anti-tumor effects (involving improving inflammation as well as oxidative stress), inhibiting pathogen infections (involving regulating pH, promoting the secretion of immune globulin, maintaining the integrity of biofilm, etc.), regulation of lipid and glucose metabolism (involving promoting lipolysis and glycolysis, while inhibiting lipid synthesis and gluconeogenesis), promotion of uric acid excretion, and anti-skin aging. “+” and “↑” indicate increasing, upregulating, promoting, accelerating, activating, or maintaining effects of AOS, while “−” and “↓” indicate decreasing, downregulating, inhibiting, or suppressing effects of AOS. AMPs: antimicrobial peptides; SCFAs: short-chain fatty acids; AMPK: adenosine 5′-monophosphate-activated protein kinase; NF-κB: nuclear factor-κB; Akt: protein kinase B; IL-1β: interleukin-1β; IL-6: interleukin-6; IL-8: interleukin-8; IL-10: interleukin-10; IFN-γ: interferon-γ; COX-2: cyclooxygenase-2; ROS: reactive oxygen species; MDA: malondialdehyde; SOD: superoxide dismutase; GSH: glutathione; CAT: catalase; STX: Shiga toxin; ST: heat-stable toxin; LT: heat-labile toxin; LPS: lipopolysaccharide; LDL: low-density lipoprotein; TC: total cholesterol; TG: triacylglycerol; FFAs: free fatty acids; AhR: aryl hydrocarbon receptor; SMCT: sodium-coupled monocarboxylate transporter; URAT1: urate transporter 1; GLUT9: glucose transporter 9; ABCG2: ATP-binding cassette superfamily G member 2; ABCG5: ATP-binding cassette superfamily G member 5; PYGL: phosphorylase glycogen lyase; PCK2: phosphoenolpyruvate carboxykinase 2; Fasn: fatty acid synthase; ACC1: acetyl-CoA carboxylase 1; SIRT1: sirtuin 1; Srebp1: sterol responsive element-binding protein 1; PGC1α: peroxisome proliferator-activated receptor γ coactivator 1α; chREBP: carbohydrate response element-binding protein; G6pase: glucose-6-phosphatase; GSK3α: glycogen synthase kinase 3α; GSK3β: glycogen synthase kinase 3β; GYS2: glycogen synthase 2; HK2: hexokinase 2; PFK1: phosphofructokinase 1.

The human gut microbiota mainly consists of Firmicutes, Bacteroidetes, Actinobacteria, and Proteobacteria at the phylum level. At the genus level, *Bacteroides*, *Clostridium*, *Fusobacterium*, *Eubacterium*, *Ruminococcus*, *Peptococcus*, *Peptostreptococcus*, *Lactobacillus*, and *Bifidobacterium* are the predominant genera among others [50,51]. Since the gut microbiota has important local and systemic impacts on our bodies, it has become increasingly attractive to modulate its composition to enhance host metabolic, immunological, and physiological functions. The influences of AOS on gut microbiota and the corresponding mechanism reported in the literature, together with the subsequent impacts on health, are listed in Table S1 and illustrated in Figure 2.

### 3.1. Promoting the Growth of Probiotics

Probiotics, as health-beneficial microorganisms, are defined by the World Health Organization as “live microorganisms which, when administered in adequate amounts, confer a health benefit on the host” [52]. They perform a variety of functions, such as maintaining gut barrier function, antioxidation, immunoregulation, antipathogen activity, anticancer effects, regulating metabolism, developing mucosal homeostasis, and even protecting the nervous system [53]. A growing number of studies have proved that AOS can regulate the diversity of gut microbiota, enriching probiotics such as *Akkermansiaceae*, *Lactobacillaceae*, *Muribaculaceae*, *Ruminococcaceae*, and many others.

*Akkermansia muciniphila* is widely known to attenuate the damage to the intestinal mucosa, protect against low-grade inflammation, restore gut barrier function, and improve insulin resistance [54,55]. Therefore, AOS is believed to ameliorate obesity-related metabolic abnormalities through increasing the abundance of the *Akkermansia* genus (e.g., *A. muciniphila*) [56,57]. Moreover, several studies have found AOS promotes certain probiotics from *Bifidobacterium*, *Faecalibaculum*, *Lactobacillaceae*, *Ruminococcaceae*, and other families, as well as their health-beneficial metabolites, e.g., ascorbic acid and glutathione (GSH) [58,59,60,61,62]. These bacteria participate in repairing oxidation- or inflammation-caused damage of not only the intestine but also organs like the liver and the kidney [22,63]. Importantly, Zhang et al. proved that AOS supplementation recovered gut microbiota dysbiosis by elevating the abundance of *Muribaculaceae*, *Ruminococcaceae*, and *Rikenellaceae* in DSS-treated colitis mice [61].

### 3.2. Inhibiting the Proliferation of Pathogenic Bacteria

Conversely, pathogenic bacteria harm the hosts, not only persistently staying in gut tracts, but also releasing bacterial toxins, such as Shiga toxin (STX), heat-labile toxin (LT), heat-stable toxin (ST), lipopolysaccharide (LPS), and others [64,65]. These toxins break gut barriers and further unbalance the normal diversity of the gut microbiota, which are produced by *Peptostreptococcaceae*, *Parabacteroides*, and the *Escherichia* and *Shigella* from the Proteobacteria [56,66,67,68]. For instance, the expansion of Proteobacteria indicates gut microbiota disorders and epithelial dysfunction, and thus is considered a marker of microbiota instability [67], whereas AOS functions as a therapeutic agent or nutraceutical against intestinal inflammatory injuries, thoroughly inhibiting the proliferation of detrimental bacteria *Helicobacteraceae* and *Peptostreptococcaceae* via the DSS-induced colitis mouse model [61]. In another study by Li et al., AOS supplementation lowered the relative abundance of harmful bacteria Proteobacteria and Fusobacteria, thereby upregulating antioxidant resistance and downregulating the inflammatory expression [69]. Additionally, AOS remarkably decreases the pathogenic *Staphylococcus* and reduces the abundance of *Enterobacteriaceae*. The latter is known as the source of virulence factors and antimicrobial resistance genes, contributing to reducing inflammatory responses and improving intestinal mucosal barrier function [32,70,71].

### 3.3. Adjusting the Firmicutes/Bacteroidetes (F/B) Ratio

The Firmicutes/Bacteroidetes (F/B) ratio represents the relative abundance of the two dominant bacterial phyla in the mammalian gut [72]. Firmicutes primarily consist of gram-positive bacteria (e.g., *Bacillus*, *Clostridium*, *Enterococcus*, *Lactobacillus*, and *Ruminococcus*), while Bacteroidetes are mainly gram-negative bacteria (e.g., *Alistipes*, *Bacteroides*, *Parabacteroides*, and *Prevotella*) [51,68]. The F/B ratio imbalance generally represents intestinal dysbiosis (e.g., IBD with a decreased ratio) or metabolic dysbiosis (e.g., obesity with an increased ratio) [72].

Specifically, obesity is generally associated with a higher F/B ratio and is significantly correlated with *Blautia hydrogenotrophica*, *Coprococcus catus*, *E. ventriosum*, *R. bromii*, and *R. obeum*, all of which belong to Firmicutes, while in lean individuals, *B. faecichinchillae* and *B. thetaiotaomicron*, belonging to Bacteroidetes, are usually enriched [73]. Research has shown that AOS intervention reduced the abundance of *Clostridia* from Firmicutes and increased that of *Bacteroidia* from Bacteroidetes, thereby lowering the F/B ratio [74]. Similarly, in the spontaneously hypertensive model rats, AOS treatment was reported to decrease the abundance of *Acidaminococcaceae* from Firmicutes; concurrently, the families within Bacteroidetes, namely *Bacteroidaceae*, *Muribaculaceae*, and *Rikenellaceae*, were amplified [66].

Conversely, Lu et al. demonstrated that MAOS significantly increased the overall F/B ratio in colitis mice. At the genus level, the results showed an increased abundance of beneficial bacterial genera like *Akkermansia* (belonging to Verrucomicrobia), *Bacteroides* (belonging to Bacteroidetes), *Dubosiella*, *Lactobacillus*, and *Ligilactobacillus* (these three belonging to Firmicutes), alongside a decrease in the conditional pathogenic bacteria *Staphylococcus*. Furthermore, the alpha-diversity was significantly elevated, as indicated by an increased Chao 1 index, Shannon index, Simpson index, and ACE index [32]. Interestingly, the effect of AOS on modulating gut microbiota composition appears to be distinctive. Han et al. compared the pig fecal microbiota-fermented AOS with other two fermented oligosaccharides (agarose oligosaccharides and κ-carrageenan oligosaccharides), and the AOS greatly increased the abundance of Bacteroidetes and decreased that of Firmicutes. The resulting strong reduction in the F/B ratio by AOS was markedly different from the effects of the other two oligosaccharides, suggesting its specific potential for improving gastrointestinal health and preventing gut diseases in animals and humans [75].

### 3.4. SCFAs Production

SCFAs, primarily acetate, propionate, and butyrate, are key metabolites produced by gut microbial fermentation of dietary fibers. They are crucial for maintaining gut health, serving as a primary energy source for intestinal epithelial cells (especially butyrate), strengthening gut barrier integrity, modulating host immune responses, and influencing nutrient absorption [76,77]. Beyond the gut, SCFAs can exert systemic effects, including anti-inflammatory and antioxidant actions; for instance, propionate shows anti-inflammatory potential relevant to tissue damage, while acetate contributes to systemic pools and can influence host metabolism [78,79]. Hence, SCFAs are considered critical indicators and mediators of a healthy gut environment. In recent years, both in vivo and in vitro studies have demonstrated that AOS intervention significantly enhances SCFAs production, most of which report increased concentrations of total SCFAs, together with acetate, propionate, butyrate, and valerate as the key SCFAs [57,80,81,82]. In the studies on the regulation of AOS in gut microbiota, SCFAs act as key metabolites, drawing researchers’ attention [58,83,84,85].

It has been accepted that the primary mechanism by which SCFAs are promoted by AOS is due to the enrichment of SCFAs-producing bacteria. AOS consistently promotes the abundance of major butyrate-producing bacteria, which predominantly belong to the Firmicutes, particularly the class *Clostridia* (e.g., families *Ruminococcaceae* and *Lachnospiraceae*) [19]. The subsequent increase in butyrate is particularly significant, providing essential fuel for colonocytes and supporting barrier function. AOS also facilitates the growth of bacteria known to produce acetate and propionate. For example, some members of the Bacteroidetes (e.g., the genus *Bacteroides*) are often found to be positively correlated with AOS intervention or SCFAs levels [86,87]. Other producers enriched by AOS include genera like *Alloprevotella* and families like *Muribaculaceae* [19]. In particular, *Muribaculaceae* is recognized for its role in degrading complex carbohydrates and promoting propionate production [88,89], contributing to epithelial health and potentially systemic benefits.

## 4. Mechanisms of AOS in Promoting Health Through Gut Microbiota

The mechanisms of AOS in promoting health through gut microbiota are illustrated in Figure 2, as well as showing their associations with disorders and diseases.

### 4.1. For Maintenance of Intestinal Barrier Integrity

The intestinal barrier, a complex multi-layer system, is essential for nutrient absorption while preventing the translocation of harmful substances and pathogens from the gut lumen into circulation. Its integrity relies heavily on the intestinal epithelium, particularly the tight junctions between epithelial cells, the overlying mucus layer, and a balanced gut microbiota composition [90]. Growing evidence indicates that AOS plays a significant role in maintaining and restoring intestinal barrier function. As discussed previously (Section 3.4), AOS consistently promotes SCFAs production, while SCFAs, particularly butyrate, are vital energy sources for intestinal epithelial cells and are known regulators of barrier function [91].

Direct evidence highlights AOS’s ability to reinforce the physical barrier. Studies by Wan et al. using a weaned piglet model challenged with enterotoxigenic *Escherichia coli* (ETEC) demonstrated that AOS supplementation significantly strengthens the intestinal barrier [92]. AOS treatment led to an increased abundance of the key tight junction protein occludin [92]. Similar findings reported that AOS facilitated the expression of multiple critical tight junction proteins, including claudin, Cx38, E-cadherin, occludin, and Zonula Occludens-1 (ZO-1) [21,22,32,58]. The upregulation of these proteins effectively “seals” the gaps between epithelial cells, reducing paracellular permeability and strengthening the barrier’s physical integrity. Beyond enhancing intercellular junctions, AOS also contributes to the overall structural health of the intestinal lining and functionally reduces gut leakage. In the ETEC-infected piglet model, histological analysis (PAS staining) revealed that AOS treatment promoted the recovery of jejunal structural damage inflicted by the infection [93]. This structural improvement was corroborated by functional measurements: AOS supplementation significantly decreased the serum concentrations of diamine oxidase and D-lactic acid and established biomarkers of increased intestinal permeability (“leaky gut”) and barrier injury [92]. Their reduction strongly suggests that AOS effectively alleviates the loss of barrier integrity. Additionally, the protective effects of AOS may extend to the intestinal mucus layer. It is reported that low-fiber diets can cause dysbiosis, mucus layer thinning, and eventually mucus degradation [94]. Notably, fermentable fibers like AOS can reverse such disorders. On the contrary, increased intestinal permeability due to barrier damage can allow harmful substances to leak through, subsequently triggering inflammation and oxidative stress.

### 4.2. For Antioxidation

As a definite modulator of gut microbiota, AOS exerts antioxidant effects through several intricate mechanisms. Primarily, the release of oxidative stress and repair of intestinal damage are attributed to increasing the abundance of probiotics, such as *Bifidobacterium*, *Roseburia*, and *Akkermansia*, together with SCFAs production [87]. AOS improves oxidative stress in a kidney-damaged model by increasing the levels of superoxide dismutase (SOD) and CAT and reducing the levels of malondialdehyde (MDA) by increasing the abundance of *Lactobacillus johnsonii* and *Lactobacillus reuteri* [61]. Serving as SCFAs, acetic, propionic, and butyric acids have been proven to exert antioxidant abilities, especially butyric acid, which not only activates the Nrf2 signaling pathway but also suppresses histone deacetylases (HDACs) to alleviate oxidative damage [95,96,97]. Moreover, SCFAs maintain an acidic intestinal environment, inhibiting the growth of harmful bacteria and reducing oxidative stress sources.

Moreover, studies have revealed that AOS influences beneficial microbiota *Faecalibacterium*, *Lactobacilli*, and *Veillonella* to secrete endogenous antioxidants like GSH and SOD, effectively scavenging ROS [21,83]. These probiotics also contribute to the metabolism of polyphenols, vitamins, and other antioxidant compounds. AOS further suppresses the proliferation of endotoxin-producing bacteria, such as *E. coli* and *Salmonella*, thereby reducing the release of pro-inflammatory factors like lipopolysaccharides, which can trigger NADPH oxidase-mediated ROS generation [61]. 

Additionally, AOS enhances the expression of tight junction proteins in intestinal epithelial cells, improving gut barrier integrity and reducing intestinal permeability, as discussed above. This prevents the translocation of endotoxins and oxidative byproducts (such as MDA) into the bloodstream, thereby mitigating systemic oxidative stress [83].

AOS also promotes the conversion of tryptophan into indole derivatives (e.g., indole-3-propionic acid) by gut microbiota, activating the aryl hydrocarbon receptor (AhR) pathway to further enhance antioxidant defenses. Zhang et al. demonstrated that AOS intervention in piglets significantly downregulated the expression of MDA, H_2_O_2_, and proinflammatory factors (TNF-α, IL-1β, IL-6, etc.), while upregulating the expression of CAT and total SOD in the liver, thereby alleviating hepatic dysfunction through the AhR signaling pathway [21].

### 4.3. For Dual-Regulation of Inflammation and Immune Responses

Inflammation can be broadly categorized into two types: (1) chronic inflammation, a dysregulated and persistent immune response often associated with disease, and (2) acute inflammation, a well-regulated, short-term defense mechanism against injury or infection [98,99]. In this aspect, increasing evidence shows that AOS regulates immune responses primarily by influencing gut homeostasis. As a result, AOS plays a “dual functional” role, as both an immune enhancers and anti-inflammatory agent, depending on the host’s immunological conditions.

In immunocompromised conditions (e.g., post-infection or chemotherapy), AOS has been shown to potentiate innate immunity by promoting the secretion of pro-inflammatory cytokines, such as IL-6, stimulating NF-κB signaling pathways, and thereby facilitating macrophage activation and pathogen clearance. Notably, the TLR4-dependent signaling cascades, including Akt/NF-κB and Akt/mTOR pathways, can be triggered by GAOS, as reported by Fang et al., resulting in the activation of macrophages and the initiation of the associated immunostimulatory effects [36]. Similarly, treatment with UAOS has been shown to upregulate the expression of Mucin-2 and secretory immunoglobulin A (SIgA) in the intestine, promoting CD4^+^T lymphocyte differentiation via the TLR4/MyD88/NF-κB signaling axis. This process subsequently enhances the expression of immune-related cytokines such as IL-1β, IL-6, and transcription factors like T-bet and GATA3, which collectively strengthen intestinal mucosal immunity [19]. When co-administration was combined with cyanidin-3-O-glucoside, as Li et al. demonstrated, AOS drastically elevated the secretion of SIgA and anti-inflammatory cytokines (e.g., IL-4, IL-10, IL-22) in male ICR mice (by nearly two-fold for all the indices). These effects were attributed to the positive modulation of the gut microbiota, which included the increased proliferation of beneficial bacteria (e.g., *Akkermansia*, *Lachnospiraceae*, and *Faecalibaculum*) and the suppression of harmful bacteria (e.g., *Helicobacter* and *Turicibacter*) [100].

Under hyperinflammatory conditions, such as autoimmune diseases and certain metabolic disorders, AOS exhibits anti-inflammatory effects by suppressing the excessive production of pro-inflammatory cytokines. Such action helps restore immune homeostasis. For example, AOS administration increased the abundance of *Anaerobiospirillum*, *Allobaculum*, *Dubosiella*, and *Prevotellaceae*, inhibiting the expression of pro-inflammatory cytokines, including TNF-α, IFN-γ, IL-1β, IL-6, and IL-17A, restricting the TLR4/MyD88/NF-κB signal pathway [21,92,101] and mitigating immune injury. Similar results of the stimulated signaling pathways were revealed by Liu et al., whereas the key gut microbiota included the upregulated *Faecalibacterium* and *Veillonella* and the suppressed *Helicobacter* and *Escherichia*-*Shigella* [83].

Furthermore, Wang et al. reported that treatment with GV-971 significantly reduced the infiltration of pro-inflammatory Th1 and M1 cells in the brain, which led to the modulation of neuroimmune activity, improving neuroinflammation and alleviating cognitive impairments in Alzheimer’s disease model mice [102]. These anti-inflammation effects were associated with AOS-mediated gut microbiota alteration, i.e., enhancement of the abundance of SCFA-producing bacteria *Lactobacillus* and *Eubacterium*, together with the content of SCFAs [21]. Similarly, AOS supplement effectively inhibited the expression of the inflammatory marker genes il1b, mpx, cxcl8a, accompanying the increase in 2-hydroxybutyric acid in the plasma of adult zebrafish (*Danio rerio*) induced by a soybean meal diet [103]. Additionally, it has been reported that GV-971 promoted the key factors of SCFAs, especially propionate and butyrate, to suppress macrophage M1 polarization and subsequent severe inflammation through inactivating the MAPK signal pathway to mitigate severe acute pancreatitis [81].

### 4.4. For Anti-Tumor Effects

Gut dysbiosis disrupts the balance between beneficial and harmful bacteria, leading to immune dysregulation and chronic inflammation that promotes tumor development. Microbial metabolites may spread through systemic circulation or an “axis” (e.g., gut–brain axis, gut–hepatic axis, gut–lung axis, etc.), influencing inflammation and tumorigenesis in distant organs. Thus, gut microbiota plays a key role in tumor progression and probably serves as a novel marker for cancer pathogenesis [104]. Therefore, improving the anti-oxidative and anti-inflammatory potential of the organism by AOS is supposed to protect against cancer.

As reviewed above (in Section 3), AOS optimizes the gut microbiota structure by inhibiting pathogenic bacteria, some of which play vital roles in tumor progression. Recent evidence shows *E. coli*, *P. anaerobius*, and *B. fragilis* could promote colorectal cancer by activating Th17 cells [105], causing DNA damage and promoting TLR2/TLR4-induced intracellular levels of ROS, accompanied by cholesterol biosynthesis and proliferation [106]. Moreover, the abundant presence of *Fusobacterium* (especially *F. nucleatum*), *Peptostreptococcus*, *Parvimonas*, and *Porphyromonas* (as commensals of the oral cavity in normal conditions) indicates metastasis of regional lymph nodes and tumor localization in the colorectal colon. Notably, *F. nucleatum* attacks and invades epithelial cells with FadA adhesion molecules that bind E-cadherin on the cell surface and activate Wnt, resulting in internalization and activation of other inflammatory genes [107]. Moreover, bacterial toxins produced by pathogenic bacteria can cross the damaged epidermal barrier, subsequently aggravating the oxidative stress and inflammatory response. Specifically, *E. coli* produces colibactin to potentiate colorectal cancer, while the toxin produced by the enterotoxigenic *B. fragilis* is related to colorectal tumors [108,109].

AOS also enhances the proliferation of SCFA-producing probiotics, which can help prevent cancer, especially butyrate, which exhibits important functions in the physiology of the host. AOS can be considered to contribute to maintaining the barrier function in epithelial cells by promoting butyrate production, thereby suppressing carcinogenesis and protecting against colon cancer. Butyrate is the main source of energy for the epithelium of the colon and has been illustrated to inhibit cancer cell proliferation, stimulate epithelial neoplastic cell line differentiation, and promote the phenotype conversion from neoplastic to non-neoplastic [94]. Moreover, butyrate has been reported to bind G proteins implicated in tumor suppression and induce Treg cells as a histone deacetylase inhibitor [94].

### 4.5. For Inhibition of Pathogen Infections

AOS inhibits pathogen infection through multiple mechanisms. One of the primary factors is that AOS contributes to reducing the abundance of pathogenic bacteria, and thus decreases the threat to the gut. AOS can act against pathogenic bacteria (e.g., *E. coli* and *S. aureus*) and pathogenic fungi (e.g., *A. niger*) [71,92,110]. *S. aureus*, which affects lipid raft-dependent sorting of sucrase-isomaltase [111], together with another life-threatening pathogen, methicillin-resistant *S. epidermidis* (MRSE), are reported to be especially effectively inhibited by AOS [112]. Similarly, Yan et al. revealed that SAOS intervention could considerably increase the production of cecum *S. enteritidis*-specific IgA and maintain it at a high level, enhancing mucosal immunity and further reducing the colonization of *S. enteritidis* in the cecum [113].

Beyond inhibiting pathogenic bacteria, AOS can promote the production of biosurfactants, antimicrobial peptides (AMPs), SCFAs, organic acids, and other metabolites by probiotics. The biosurfactants can disrupt the integrity of pathogen cell membranes and interfere with their ability to adhere to host tissues and form resilient biofilms, showing significant antimicrobial, anti-adhesive, and anti-biofilm capabilities [114]. AOS can also stimulate antimicrobial peptides, including the potent antimicrobial bacteriocins, leading to leakage of intracellular contents and cell death [115].

Moreover, except for SCFAs, AOS can promote various organic acids like ascorbic acid and lactic acid. Mi et al. demonstrated that AOS pretreatment elevated the level and facilitated the colonization of SCFA-producing bacteria *Bacilli*, *Bifidobacterium*, *Dubosiella*, *Eggerthellaceae*, *Eubacterium*, *Faecalibaculum*, *Romboutsia*, and *Turicibacter*, and further elevated the level of metabolites and SCFAs, especially acetate and valerate [58]. The production of these acidic metabolites lowers the intestinal pH, creating an environment unfavorable for the growth of pH-sensitive pathogens like *Escherichia* and *Salmonella*. Moreover, AOS can stimulate *Lactobacillus* to produce hydrogen peroxide, which damages the biofilm formation of *H. pylori* and prevents intestinal and urogenital infection [116,117]. It is to be noted that *Lactobacillus* and other lactic acid bacteria (LAB) can inhibit pathogen quorum sensing (QS) systems [118], which are critical for biofilm formation and virulence in pathogens like *P. aeruginosa* and *S. aureus*. Specifically, *L. plantarum* is known to interfere with QS signaling molecules and biofilm formation, expressing inhibitory effects on pathogen growth in murine burn wound models [118]. Therefore, AOS appears to be a promising pathogenic infection inhibitor in multiple ways.

### 4.6. For Regulation of Lipid and Glucose Metabolism

Lipid metabolism dysregulation, including lipid accumulation, abnormal lipid biosynthesis and degradation, and lipid oxidation, serves as the primary cause of metabolic diseases, while glucose metabolism disorders usually lead to insulin resistance and diabetes mellitus. AOS has shown significant potential in modulating lipid metabolism and glucose homeostasis. It has been shown that AOS via *Bacteroides* promotes the production of citric acid, fumaric acid, and α-ketoglutaric acid to accelerate the tricarboxylic acid cycle (TCA cycle) to improve energy homeostasis, and upregulates the level of hydroxy fatty acids (FAHFAs) to improve glucose homeostasis, which are respectively described as the *Bacteroides acidifaciens*–FAHFAs axis and *Bacteroides*–TCA cycle axis by Yan et al. [119]. Moreover, studies have demonstrated that AOS can activate the phosphatidylinositol 3-kinase/Akt/glycogen synthase kinase 3β (PI3K/Akt/GSK3β) signaling pathway, upregulate the expression of hepatic low-density lipoprotein receptor (LDLR), and downregulate hepatocyte nuclear factor-1α (HNF-1α) to lower the expression of proprotein convertase subtilisin/kexin type 9 (PCSK9). This cascade reduces the degradation of LDLR, promotes the uptake of low-density lipoprotein (LDL) by liver cells, and subsequently lowers the plasma LDL–cholesterol (LDL-C) level [120]. Moreover, AOS facilitates lipid catabolism by enhancing PGC-1α-mediated lipophagy-FFA β-oxidation, which alleviates high-fat-induced hepatic steatosis [121]. Such a mechanism of AOS seems similar to other prebiotics like D-arabitol, of which SCFAs promote AMPK-PGC-1α-related white adipose tissue browning [122]. Additionally, AOS has been shown to upregulate the expression of key lipid metabolism genes, such as Apoa1, Apoa4, Agpat1, DGAT, MGAT, and ABCG5, which collectively increase lipase activity in the liver, facilitating fat metabolism and reducing liver fat accumulation [123].

Importantly, AOS regulates lipid metabolism through interactions with the gut microbiota. Studies have demonstrated that diets containing AOS significantly reduce the abundance of harmful bacteria, such as *Gammaproteobacteria*, while promoting the growth of beneficial species like *Cetobacterium*. This microbial modulation is associated with the inhibition of adipogenic marker overexpression (including Fasn, ACC1, SIRT1, Srebp1, and PGC1α) and the stimulation of lipid metabolism gene expression (such as Agpat 1, Apoa1, Apoa4, Apoa48, DGAT, and FBP2) in the intestine, which contribute to improve lipid homeostasis [57,69].

Furthermore, Wang et al. revealed that AOS intervention could remarkably elevate the level of probiotics *A. muciniphila*, *L. reuteri*, and *L. gasseri*, which have clinically been proven to have health-promoting effects by improving lipid and glucose metabolism. Such modulation of the microbiome promotes the production of the SCFAs acetic acid, propionic acid, and butyric acid, which collectively contribute to reducing fasting blood glucose, serum insulin levels, fat accumulation in the liver, and further improve lipid metabolism in high-fat diet-fed mice. At the same time, AOS inhibited the Fasn gene, which is critical for the de novo lipogenesis pathway, as well as other fatty acid synthase-related markers, namely ACC1, SIRT1, Srebp1, and PGC1α. As a result, AOS reduced TG and LDL-C levels and decreased body and adipose tissue weight [57]. In parallel, Li et al. demonstrated that UAOS supplementation effectively reduced fasting insulin, fasting serum glucose, insulin resistance index, and blood insulin and glucose to improve insulin resistance and impaired glucose tolerance in C57BL/6 mice fed with a high-fat diet. Such improvement was associated with upregulation of the *Lactobacillus* and *Akkermansia* and downregulation of *Bacteroides* and *Parabacteroides*, suggesting that the regulation effects of metabolic disorders for AOS were due to regulating gut microbiota [56].

### 4.7. For Promotion of Uric Acid Excretion

The gut microbiota plays a critical role in regulating hyperuricemia by modulating purine metabolism and uric acid excretion. In purine metabolism, certain probiotics, such as *L. gasseri* PA-3, have shown potential in preventing hyperuricemia by metabolizing inosine and hypoxanthine, thereby reducing the intestinal absorption of purines. Furthermore, certain species within *Lactobacillus* have been reported to alleviate hyperuricemia in rats by inhibiting xanthine oxidase activity [124].

Recent studies have suggested that the facilitation of uric acid excretion is the primary mechanism by which AOS contributes to the alleviation of hyperuricemia. Wei et al. reported that AOS intervention altered the gut microbiota composition, decreasing the abundance of bacteria such as *Bilophila*, *Tuzzerella*, *Bryobacter*, and *Comamonas*. In contrast, the increased bacteria mainly contained *Muribaculum*, *Ruminococcus*, *Faecalibaculum*, *Clostridia_UCG-014*, *Lachnospiraceae_UCG_006*, and *Lachnospiraceae_NK4A136_group*. As a result, renal uric acid transporters are regulated, including the downregulation of URAT1, SMCT, and GLUT9, as well as the upregulation of ABCG2. Along with these, the levels of proinflammatory cytokines like IL-1β, IL-12, and IL-18 in the kidney were decreased, leading to a lowered serum uric acid level and lowered blood urea nitrogen, promoting uric acid excretion [82,125].

### 4.8. For Anti-Skin Aging

Skin aging is associated with diversity reduction and structure changes in the gut microbiota, of which it has been reported that older adults usually show an increase in Bacteroidetes and *Enterobacteriaceae* and a decrease in *Bifidobacterium*, *Faecalibacterium prausnitzii*, *Clostridium cluster XIV*, and others [126]. A report by Gao et al. indicated that AOS (HAOS, GAOS, and MAOS), especially MAOS, evidently performed an outstanding skin improvement effect in aging model mice. The resulting *Lachnospiraceae*, *Butyricicoccus*, and *Butyricimonas*, connected to butyrate production, were associated with gut–skin axis (colonic butyrate–HIF-1α axis) homeostasis, which was mediated by AOS. By balancing mitochondrial metabolism and maintaining a necessary mitophagy level, AOS contributed to improving the skin stratum corneum water content, the skin elasticity, the epidermal thickness, and the tortuosity of the dermal–epidermal junction, eventually alleviating skin aging [34].

## 5. AOS in Disease Prevention and Treatment

### 5.1. Gastrointestinal Diseases

In terms of gastrointestinal diseases, such as *C. difficile* infection (CDI) and IBD, the gut microbiota plays a crucial role in regulating intestinal function and inflammation. Dysbiosis, characterized by an increased F/B ratio, leads to impaired SCFAs production and inflammation. The literature has shown that AOS treatment effectively modifies the gut microbiota, decreasing pro-inflammatory bacteria like *Lachnospiraceae* and *Proteobacteria* while enhancing anti-inflammatory bacteria such as *Bacteroidetes*, potentially alleviating inflammation and improving gut health [101]. In addition to these microbiota alterations, AOS, particularly GAOS, plays a significant role in treating various gastrointestinal inflammatory diseases by modulating gut microbiota and immune responses [85]. It alleviates symptoms like weight loss, colon shortening, and inflammatory infiltration in ulcerative colitis by downregulating pro-inflammatory cytokines such as IL-6, IL-1β, TNF-α, and Cox-2, and by activating anti-inflammatory pathways like the TLR-4/NF-κB pathway. AOS treatment also promotes the growth of SCFAs-producing bacteria, such as *Ruminococcaceae* and *Bacteroidaceae*, enhancing the production of beneficial SCFAs, especially acetate, butyrate, and isovalerate. These effects contribute to the reduction in inflammation and improvement in colon health, demonstrating AOS’s potential as a therapeutic agent for IBD and chemotherapy-induced intestinal damage [85,101,127].

Moreover, a derivative of AOS, oligoguluronate lithium, exhibited considerable potential for treating ulcerative colitis. Compared with oligoguluronate sodium and lithium carbonate, the lithium-modified AOS significantly increased the diversity of intestinal microorganisms in the short term, alleviating the clinical symptoms and histopathological changes, and together reduced the levels of *Escherichia-Shigella* spp. and *Romboutsia* spp. At the same time, oligoguluronate lithium resulted in improvement of weight loss and rectal bleeding symptoms, indicating it as a promising AOS-based drug for treating ulcerative colitis [128].

In recent years, fecal microbiota transplantation (FMT) has been successfully utilized in treating gastrointestinal diseases, while AOS pretreatment of the gut microbiota enhanced the therapeutic effects. Zhang et al. investigated the different doses of AOS pretreatment of FMT for improving intestine damage and found that 10 mg/kg BW of AOS pretreatment was the best concentration to reduce the F/B ratio, increase the abundance of beneficial bacteria *Bacteroidales* and *Lactobacillaceae*, inhibit the growth of harmful bacteria *Desulfovibrionaceae*, and restore blood metabolites along with intestinal barrier maintenance [22].

### 5.2. Metabolic Diseases

#### 5.2.1. Obesity

Obese individuals exhibit higher F/B ratios and an abundance of harmful bacteria, such as *Ruminococcus* and *Lachnospiraceae*, while beneficial bacteria, such as *Bacteroides*, are reduced [129]. AOS treatment helps restore the balance by increasing beneficial bacteria, including *Lactobacillus* and *Akkermansia*. This leads to improvements in insulin resistance, glucose tolerance, and lipid metabolism, as demonstrated in obesity models [56,74].

#### 5.2.2. Diabetes

Diabetes is a chronic metabolic disorder characterized by high blood sugar levels, resulting from either insufficient insulin production or the body’s inability to effectively use the insulin it produces [130,131,132]. AOS has shown promise in managing diabetes by improving insulin sensitivity and glucose metabolism. Research indicates that AOS and its precursor regulate serum metabolites, improving insulin sensitivity and reducing fasting blood glucose levels, contributing to better glucose metabolism and improved insulin resistance. These effects are connected to the modulation of gut microbiota, with which the enriched beneficial bacteria contain *Faecalibacterium*, *Akkermansia*, and *Lactobacillus*, while the decreased harmful bacteria include *Desulfovibrio* and *Blautia*. In the hyperglycemic zebrafish model, AOS significantly reduced blood glucose levels by promoting glucose oxidation and suppressing lactate production. AOS also enhances SCFAs production, including acetic, propionic, and butyric acid, which helps lower blood glucose and improve insulin sensitivity in diabetic models [56,133,134].

#### 5.2.3. Hyperuricemia

Hyperuricemia is a metabolic disorder caused by purine metabolism disorder or uric acid excretion disorder. In clinical findings, an increase in *Prevotella*, *Bacteroides*, *Barnesiella*, and *Parasporobacterium* and a decrease in *Faecalibacterium*, *Coprococcus*, and *Alistipes* were found in gout patients [135,136]. Additionally, gout patients’ gut microbiomes show a deficiency in microbes carrying the allantoinase gene (which converts uric acid to urea) but an abundance of microorganisms with the xanthine dehydrogenase gene [135,137]. Méndez-Salazar et al. compared the taxonomic composition of the gut microbiota between gout patients with and without tophi. They found that compared to gout patients with tophi, those without tophi exhibited a greater abundance of *Ruminococcus*, *Akkermansia*, *Bacteroides*, and *Phascolarctobacterium* in the gut, whereas those patients with tophaceous gout showed a further increase in the abundance of *Escherichia*, *Shigella*, *Sarcina*, *Rikenellaceae*, and *Lachnospiraceae* [138]. These changes are associated with reduced production of SCFAs and alterations in purine metabolic pathways.

The present therapies utilize uric acid-lowering medications, xanthine oxidase (XOD) inhibitors (e.g., allopurinol and febuxostat), and uricosuric agents (e.g., benzbromarone). However, in clinical practice, caution is advised when administering anaerobic antibiotics to patients with hyperuricemia or gout, as they may disrupt the functions of the intestinal microbiota [139]. While there are specific bacterial strains engaged in uric acid metabolism, biotherapeutic agents composed of uric acid-degrading bacteria hold promise for the treatment of hyperuricemia and gout, becoming a new prospect.

#### 5.2.4. Hypertension

With 1.28 billion adult patients, hypertension has become a major cause of premature death worldwide, which often accompanies stroke, heart failure, and other cardiovascular diseases [140,141]. Modern studies show that gut microbiomes can regulate blood pressure through several mechanisms. Specifically, the gut microbiota and its metabolites, or FMT, can effectively improve cardiovascular diseases such as hypertension [140,142,143]. At the same time, propionate can alleviate cardiovascular damage caused by hypertension [143].

Mushtaq et al. indicated that the F/B ratio was higher in hypertensive patients at the phylum level [144]. It has been recorded that the abundance of *Prevotella_9*, *Klebsiella*, *Megasphaera*, *Parasutterella*, and *Escherichia-Shigella* was increased in patients with hypertension, while the abundance of *Bacteroides* and *Faecalibacterium* decreased in hypertensive patients at the genus level [144,145]. Han et al. adopted the spontaneously hypertensive rat model to show that potassium AOS treatment significantly lowered the F/B ratio by nearly one half compared to the vehicle-treated group [66]. Moreover, this intervention significantly increased the relative abundance of *Bacteroidales_S24-7_group*, *Rikenellaceae*, and *Bacteroidaceae* of *Bacteroidetes* and decreased the relative abundance of *Acidaminococcaceae* of *Firmicutes* at the family level, and downregulated the relative abundance of *Phascolarctobacterium* and *Prevotella* at the genus level, which may be beneficial to improve blood pressure and decrease LPS metabolites in the spontaneously hypertensive rat model [66].

### 5.3. Anti-Cancer

AOS has demonstrated promising anti-tumor properties in various experimental models, exerting its anti-cancer effects through multiple mechanisms by modulating the gut microbiota, restoring immune homeostasis, and inhibiting the proliferation/migration of tumor cells. In patients with colorectal cancer (CRC), the gut microbiome can directly or indirectly affect CRC by secreting metabolites, invading tissues, and modulating host immune response [146]. *Clostridium*, *Peptostreptococcus*, *Porphyromonas*, *Prutella*, and *Bacteroides* are the most significant bacteria associated with CRC [146,147,148]. In terms of leukemia, AOS (by enzymatic degradation, DP = 20–24, dose = 500 μg/mL) enhances defense mechanisms, upregulating the synthesis of cytotoxic cytokines in human mononuclear cells [149].

To be noted, the anti-cancer activity of AOS depends on its DP and M/G ratio. For DP, Chen et al. showed that only AOS with DP 5 resulted in an increase in antioxidant activity in osteosarcoma patients, and it significantly inhibited the growth of osteosarcoma cells [26]. In the research focusing on the bioactivity of MAOS and GAOS with DP ranging from 3 to 9 (i.e., MAOS3–9 and GAOS3–9) in relation to stimulating cytokines in RAW264.7 cells, MAOS with DP 7 (M7) and GAOS with DP 8 (G8) were the most effective oligomers, and among MAOS, M7 and G8 were suggested to be the most suitable molecular size and structural conformation to serve as ligands of cellular receptors for cytokine secretion [27].

AOS derivatives exert significant anti-tumor activity by immunomodulation, especially for the sulfated derivatives of AOS, which are widely reported as immunomodulators that exhibit stronger anti-cancer activity than the original AOS [18,150]. For instance, according to Hu et al., the AOS sulfate derivative (molecular weight = 3.8 kDa, sulfation degree = 1.3, dose = 100 μg/kg) induces tumor regression indirectly by modulating the host’s immune responses [151].

### 5.4. Neurodegenerative Diseases

The most prevalent and serious neurodegenerative diseases in the world include Alzheimer’s disease and Parkinson’s disease. As a progressive neurodegenerative disease, Alzheimer’s disease is characterized by neuritic plaques formed and neurofibrillary tangles in the brain of affected individuals. There are two major factors widely accepted: (1) nerve cell damage induced by oxidative stress, associated with the aging process and various neurodegenerations; and (2) Aβ accumulation and the resulting neurotoxicity [152]. Cumulative evidence has demonstrated that gut microbiota imbalance is associated with increased differentiation and proliferation of peripheral T cells, increased infiltration of pro-inflammatory T cell subtypes into the brain, activated pro-inflammatory microglia subtypes, and the appearance of Aβ plaques and other markers of Alzheimer’s disease pathology [153]. Interestingly, AOS has been found not only to effectively prevent neurotoxicity induced by oxidative stress and protect NT2 neurons from death, but also to inhibit Aβ formation in the Alzheimer’s disease model [153].

Recent studies have discovered that probiotics produce various essential neurotransmitters and specific neuromodulators, e.g., *Lactobacillus* spp. and *Bifidobacterium* spp.: gamma-aminobutyric acid (GABA); *Escherichia* spp., *Bacillus* spp., and *Saccharomyces* spp.: noradrenalin; *Candida* spp., *Streptococcus* spp., *Escherichia* spp., and *Enterococcus* spp.: serotonin; *Bacillus* spp.: dopamine; *Lactobacillus* spp.: acetylcholine [154]. Importantly, *Lactobacilli reuteri* induce oxytocin accumulation, while oxytocin is known to participate in buggering stress-induced mental and physical impairments, and thus believed to improve tissue regeneration [155,156]. Along with this, other gut *Lactobacilli* were revealed to produce neuroactive molecules, including catecholamines and GABA [157,158]. Therefore, the probiotic promotion function of AOS suggests it as a novel neuroprotective supplement. Besides, Bi et al. reported that Se-modified MAOS exhibited neuroprotection effects through antioxidation and anti-inflammation, extending the applications of AOS derivatives towards neurodegenerative diseases [159,160].

Wang et al. revealed that the development of Alzheimer’s disease is associated with changes in the gut microbiota and immune cell infiltration, and the gut microbiota is necessary for immune cell infiltration and microglial activation in the brain [102]. Additionally, GV-971 administration can restrain the development of Alzheimer’s disease by increasing the relative abundance of *Bacteroides* and Verrucomicrobia to inhibit the associated phenylalanine/isoleucine accumulation, improve associated neuroinflammation, and reverse cognitive impairment in Alzheimer’s disease model mice [102]. Furthermore, Bosch et al. illustrated that supplementing GV-971 at the beginning of Aβ deposition or after Aβ deposition has reached a higher level can target the microbiome–microglia–amyloid axis, improving Aβ plaque pathology and alleviating neuroinflammation in a sex-dependent manner, providing new insights into the pathogenesis and treatment of Alzheimer’s disease [161].

### 5.5. Improving Reproductive Health

Some studies have shown that AOS is qualified with the potential to improve host reproduction by regulating gut microbiota and its metabolites. Zhou et al. found that sperm metabolism was associated with the increased bacteria *Enterobacter* and the decreased harmful bacteria such as *Streptococcus*, *Prevotellaceae_UCG-001*, and *Prevotellaceae_NK3B31_group* in the intestine, which can be adjusted by AOS [162], moreover facilitating expression of the sperm-related proteins, such as CatSper 8, PKA gelsolin, and Zn-α-2 glycoprotein, which were regulated by the beneficial bacteria *Bifidobacterium*, *Coprococcus*, and *Butyricicoccus* [163,164]. In addition, these important proteins can promote sperm motility and quality. In parallel, Zhao et al. showed that AOS treatment could relieve the busulfan-induced reproductive ability damage in males by improving the intestinal microenvironment, which was mediated by raising the level of beneficial microbiota *Bacteroidales* and *Lactobacillaceae* and suppressing the proliferation of harmful bacteria *Desulfovibrionaceae* [165].

### 5.6. Other Diseases

Patients with acute pancreatitis often exhibit significant gut microbiota dysbiosis and intestinal barrier disruption. The intestinal environment in these individuals is typically characterized by an overrepresentation of harmful bacteria such as *Escherichia-Shigella*, *Enterococcus*, and certain members of the *Enterobacteriaceae* family. Concurrently, there is a marked reduction in SCFAs and beneficial bacteria, including *Prevotella_9*, *Faecalibacterium*, *Blautia*, and *Bifidobacterium* [166,167]. These alterations contribute to increased intestinal permeability, systemic inflammation, and disease progression. Additionally, Chen et al. showed that GV-971 (400 mg/kg) possessed the potential to modulate gut microbiota composition, enhance SCFAs production, and reinforce the mucosal barrier, thus offering a promising therapeutic strategy for mitigating intestinal damage and inflammation in acute pancreatitis [81].

In addition, AOS has demonstrated protective effects against osteoporosis, particularly age-related bone loss. According to Zhang et al., AOS (molecular weight = 4.9 kDa, M/G = 0.53) improved gut microbiota and bile acids metabolism by increasing the abundance of intestinal bacteria *Bifidobacterium* and *Clostridium*. Consequently, it elevated the levels of bile acids isoLCA and isoalloLCA, inhibiting the intestinal lamina propria Th17 cell subsets differentiation and alleviating the peripheral inflammation to improve osteosarcopenia induced by estrogen deprivation, including bone loss and muscle hypofunction [168]. *Bifidobacterium* and *Clostridium* exhibited excellent performance in osteosarcopenia [169,170]. Some studies have proved that Th17 cell subsets are the key factors in osteoclastogenesis and bone loss through upregulating the expression of pro-inflammatory cytokines (IL-17, TNF-α, etc.) while downregulating the expression of anti-inflammatory factors (IL-10, etc.) [171,172]. Additionally, receptor activator of nuclear factor-κB ligand (RANKL) induced osteoclastogenesis, bone damage, and bone loss, while osteoprotegerin (OPG) binds to RANKL to inhibit RANKL, combining with receptor activator of nuclear factor-κB (RANK), so that these conditions are reversed [173]. In another study, AOS has been demonstrated to express protective effects against osteoporosis, particularly age-related bone loss in such a mechanism, in which AOS promoted the production of OPG to suppress the binding of RANK and RANKL to reduce osteoclast differentiation and activity, ultimately helping to prevent bone resorption and maintain bone homeostasis [174].

## 6. Limitations and Perspectives

Significant progress has been achieved in exploring AOS and its interaction with gut microbiota. Yet, limitations in microbiota-centered research fields remain to be further addressed. One critical concern is that there are no unified quality control standards for AOS at the present stage. Therefore, AOS with varied purity, structures (e.g., DP, M/G ratio, end glycosyl structure, etc.), and preparation methods expresses distinctive interactions with gut microbiota. For example, AOS with a molecular weight of 0.8 kDa is more easily fermented than that of 0.3 kDa [175]. Moreover, the interaction of AOS and gut microbiota considerably depends on individual differences, such as genetics, diet, lifestyle, environment, and others; therefore, individual-based treatment is of great necessity. At the same time, the interindividual microbiome variability is also worthy of investigation, as it significantly influences the responsiveness to AOS-based interventions. Targeted microbiome therapeutic interventions are likely to be provided, for instance, by targeting butyrate-producing probiotics or inhibiting pathogenic bacteria in combination with personalized microbiota-based interventions. Such an approach can involve employing microbiota profiling via metagenomics and metabolomics to identify deficiencies in specific microbial imbalances, and nutritional and therapeutic strategies may be achieved by using modified AOS.

Another concern of AOS is safety, although current human trials on AOS, including GV-971 for Alzheimer’s disease and OligoG CF-5/20 for cystic fibrosis, have demonstrated its safety and the tolerance of AOS, which informs dosage selection [39,176]. Animal and cell experiments also prove that AOS is safe, with no treatment-related adverse events at the administration doses [49,61,69,127]. Nevertheless, long-term safety and potential risks under varying physiological or pathological conditions warrant further investigation.

Notably, the effects of AOS on gut microbiota will be evaluated by not only characterizing taxonomic features (i.e., diversity, composition, and population) but also the distinctive metabolite profiles. Since gut microbiota produce both beneficial compounds (e.g., SCFAs and other organic acids) and toxins (e.g., LT, ST, and STX) in a global view, the approach of “AOS–gut microbiota interaction metabolomics” may be more direct and functional for investigating gut microbiota improvement. The developed metabolic biomarkers will refine efficacy assessments and guide precise optimizations of AOS interventions.

In conclusion, deeper exploration into the interactions between AOS and gut microbiota enhances our understanding of AOS–microbiota–health relationships. Examining this key aspect not only contributes to the search for precise intestinal nutrition strategies but also accelerates the development of targeted functional food or medicinal products to promote well-being and prevent diseases.

## Supplementary Materials

The following supporting information can be downloaded at https://www.mdpi.com/article/10.3390/nu17121977/s1; Table S1: Influences of AOS on Gut Microbiota associated with beneficial functions. References [177,178,179,180,181,182,183,184,185] are cited only in Supplementary Materials.

## Abbreviations

The following abbreviations are used in this manuscript:

4-HNE	4-hydroxynonenal
AhR	Aryl hydrocarbon receptor
ALT	Alanine aminotransferase
AMPs	Antimicrobial peptides
AOS	Alginate oligosaccharide
APP	Amyloid precursor protein
AST	Aspartate aminotransferase
Aβ	Amyloid-β
CDI	*Clostridium difficile* infection
CRC	Colorectal cancer
DP	Degree of polymerization
DSS	Dextran sodium sulfate
ER	Endoplasmic reticulum
ETEC	Enterotoxigenic *Escherichia coli*
F/B	Firmicutes/Bacteroidetes
FFAs	Free fatty acids
FMT	Fecal microbiota transplantation
G	α-L-guluronic acid
GABA	Gamma-aminobutyric acid
GAOS	Oligoguluronate alginate oligosaccharide
GSH	Glutathione
HAOS	Heterogeneous alginate oligosaccharide
HDAC	Histone deacetylase
IBDs	Inflammatory bowel diseases
LDLR	Low-density lipoprotein receptor
LPS	Lipopolysaccharide
LT	Heat-labile toxin
M	β-D-mannuronic acid
MAOS	Oligomannuronate alginate oligosaccharide
MDA	Malondialdehyde
MRSE	Methicillin-resistant *Staphylococcus epidermidis*
NMPA	National Medical Products Administration
Nrf2	Nuclear factor erythroid 2-related factor 2
OPG	Osteoprotegerin
QS	Quorum sensing
RANK	Receptor activator of nuclear factor-κB
RANKL	Receptor activator of nuclear factor-kB ligand
ROS	Reactive oxygen species
SAOS	Saturated alginate oligosaccharide
SCFAs	Short-chain fatty acids
SOD	Superoxide dismutase
ST	Heat-stable toxin
STX	Shiga toxin
TC	Total cholesterol
TG	Triacylglycerol
TNF-α	Tumor necrosis factor
UAOS	Unsaturated alginate oligosaccharide
XOD	Xanthine oxidase
ZO-1	Zonula Occludens-1
