# Supplementary material for: Alginate Oligosaccharide and Gut Microbiota: Exploring the Key to Health
Source: Nutrients. 2025 Jun 11;17(12):1977. doi: 10.3390/nu17121977 (PMC12196054; doi:10.3390/nu17121977)
Supplement: Supplementary file 1 [file nutrients-17-01977-s001.zip › nutrients-3678412-supplementary.pdf]

Alginate Oligosaccharide and Gut Microbiota: Exploring the Key to Health

Meiling Song, Lin Chen, Chen Dong, Minghui Tang, Yuan Wei, Depeng Lv, Quancai Li and Zhen Chen

Supplementary Material

Table S1. Influences of AOS on Gut Microbiota associated with beneficial functions.

Influenced Gut microbiota			Functions & Therapeutics	Involved Proteins/ Cytokine/Pathway	Changes of Metabolites	Reference
Family	Genus	Species				
**Firmicutes (Bacillota)**						
Lactobacillaceae (+)	*Lactobacillus* (+)	*L. rhamnosus*<br>*L. gasseri*<br>*L. reuteri*<br>*L. johnsonii*	Intestinal barrier function (+)<br>Lipid metabolism (+)<br>Glucose metabolism (+)<br>Metabolic syndrome (−)<br>Neuroprotection (+)<br>Inflammation (−)<br>Oxidative Stress (−)<br>IBD (−)	Nrf2 (+)<br>AhR (+)<br>TLR4 (−)<br>GSH (+)<br>SOD (+)<br>CAT (+)<br>MDA (−)<br>TG (−)<br>LDL-C (−)<br>TNF-α(−)<br>IL-1β(−)<br>IL-6 (−)	Acetate (+)<br>Propionate (+)<br>Butyrate (+)<br>Valerate (+)	[1–6]
	Ligilactobacillus (+)		Oxidation (−)			
Lachnospiraceae (+)	*Dorea*		growth performance (+)	Ghrelin	SCFAs (+)	[7]
	Lachnoclostridium (+)<br>*Roseburia* (+)		Immunity (+)			[8]
	Coprococcus (+)	*C. catus*	Reproduction (+)	CatSper (+)<br>PKA gelsolin (+)<br>zinc-α2-glycoprotein (+)		[9]
	Blautia (−)		Lipid metabolism (+)<br>Diabetes (−)<br>Insulin resistance (−)<br>Inflammation (−)		LPS (−)	[3,10,11]
Leuconostocaceae	*Leuconostoc*	*L. mesenteroides*	IBD (−)		Blood metabolites (+)	[12]
Ruminococcaceae (+)	*Ruminococcus*<br>*Faecalibacterium (+)*	*R. albus*<br>*F. prausnitzii*	Immunity (+)<br>Gut barrier function (+)<br>Uric acid excretion (+)<br>Neuroprotection (+)<br>Intestinal mucosal damage (−)<br>Intestinal inflammation (−)	TLR4/MyD88/NF-κB<br>GSH (+)<br>ABCG2 (+)<br>URAT1 (−)<br>SMCT (−)<br>GLUT9 (−)	SCFAs (+)	[8,13–16]

Influenced Gut microbiota			Functions & Therapeutics	Involved Proteins/ Cytokine/Pathway	Changes of Metabolites	Reference
Family	Genus	Species				
			Pancreatitis (−)<br>Cardiovascular disease (−)<br>Hyperuricemia (−)	TNF-α(−)<br>IL-1β(−)<br>IL-6 (−)<br>IL-12 (−)<br>IL-18 (−)		
Bacillaceae	*Butyricicoccus*		skin aging (-)		Butyric acid (+)	[17]
Bacillaceae	*Bacillus*		Neuroprotection (+)		Noradrenalin (+)<br>Acetylcholine (+)<br>Dopamine (+)	[18]
Eubacteriaceae	*Eubacterium*		Colonic mucosal immunity (+)		SCFAs (+)	[5]
Clostridiaceae	*Clostridium*	*C. difficile*	Infection (−)<br>IBD (−)			[19]
Clostridiaceae	*Clostridium*		Intestinal inflammation (−)<br>Skin aging (−)<br>Colorectal cancer (−)			[20–23]
Clostridiaceae	*Butyribacterium*	*B. methylotrophicum*			Butyrate (+)	[24]
Staphylococcaceae	*Staphylococcus* (−)	*S. aureus*<br>*S. epidermidis*	Inflammation (−)<br>Infection (−)<br>Bacteremia (−)<br>Gut microenvironment homeostasis (+)	IL-6 (−)<br>IL-10 (+)<br>TNF-α (−)		[25,26]
Enterococcaceae	*Enterococcus* (−)	*E. faecalis*<br>*E. faecium*	Infection (−)			[7,25]
Peptostreptococcaceae	*Peptostreptococcus*	*P. anaerobius*	Immunity (+)<br>Gut barrier function (+)<br>Infection (−)	TLR4 (−)<br>TNF-α(−)<br>IL-1β(−)<br>IL-6 (−)	SCFAs (+)	[27–30]
Peptostreptococcaceae	*Romboutsia* (+/−)	*R. ilealis*	Gut barrier function (+)<br>IBD (−)			
Peptoniphilaceae	*Parvimonas*		Inflammation (−)<br>Colorectal cancer (−)			[31]
Erysipelotrichaceae	*Faecalibaculum* (+)		Immunity (+)<br>Oxidation (−)<br>Inflammation (−)<br>Uric acid excretion (+)<br>Hyperuricemia (−)<br>Diabetes (−)<br>Neuroprotection (+)	ABCG2 (+)<br>URAT1 (−)<br>SMCT (−)<br>GLUT9 (−)<br>IL-1β(−)<br>IL-12 (−)<br>IL-18 (−)	Acetate (+)<br>Propionate (+)<br>Isovalerate (+)	[11,13,16,29,32–34]

**Influenced Gut microbiota**			**Functions & Therapeutics**	**Involved Proteins/ Cytokine/Pathway**	**Changes of Metabolites**	**Reference**
Family	Genus	Species				
				SOD (+)<br>CAT (+)<br>MDA (−)		
	Dubosiella (+)		Intestinal barrier function (+)<br>Gut inflammation (−)<br>Immunity (+)	IL-1β (−)<br>IL-6 (−)<br>TNF-α (−)	Acetic acid (+)<br>Propionic acid (+)<br>Valeric acid (+)	[29]
Streptococcaceae	*Streptococcus*	*S. pyogenes*	Reproduction (+)<br>Infection (−)		Serotonin	[35]
Veillonellaceae	*Veillonella* (+)		Supply energy (+)<br>Gut microenvironment homeostasis (+)<br>Inflammation (−)<br>oxidative stress (−)<br>Intestinal barrier function (+)	TLR4 (−)<br>IL-1β (−)<br>IL-6 (−)<br>TNF-α (−)<br>COX-2 (−)<br>MDA (−)<br>T-SOD (+)<br>CAT (+)	Propionate (+)	[27,33]
	Megasphaera		Hypertension (−)			[36]
Turicibacteraceae	*Turicibacter* (+/−)		Inflammation (−)<br>Immunity (+)<br>Intestinal barrier function (+)	SIgA (+)<br>IL-6 (−)<br>IL-1β (−)<br>IL-10 (+)<br>IL-22 (−)<br>TNF-α (−)	SCFAs (+)	[29,32]
**Bacteroidetes (Bacteroidota)**						
Bacteroidaceae	*Bacteroides*	*B. fragilis*<br>*B. uniformis*<br>*B. acidifaciens*	Colitis (−)<br>Glucose metabolism (+)<br>Intestinal barrier function (+)<br>Lipid metabolism (+)<br>Intestinal mucositis (−)<br>Alzheimer's disease (−)<br>Cardiovascular disease (−)<br>Hypertension (−)<br>Hyperuricemia (−)	IL-6 (−)<br>IL-10 (+)<br>IL-1β (−)<br>IL-12 (−)<br>IL-18 (−)<br>TNF-α (+)<br>MDA (−)<br>CAT (+)<br>SOD (-)<br>Th17 cell (−)<br>TLR4 (−)	Acetate (+)<br>Propionate (+)<br>Butyrate<br>Isovalerate (+)<br>*B. fragilis* toxin (−)	[2,3,6,10,15,16,25,33,34,37]
Rikenellaceae (+)	*Alistipes* (+)		Lipid metabolism (+)<br>Colitis (−)<br>Skin aging (−)	IL-1β (−)<br>IL-6 (−)	Acetate (+)<br>Propionate (+)<br>Butyrate (+)	[10,15,17,28,38]

Influenced Gut microbiota			Functions & Therapeutics	Involved Proteins/ Cytokine/Pathway	Changes of Metabolites	Reference
Family	Genus	Species				
			Hypertension (−)	IL-10 (+)<br>TNF-α (−)<br>AMPK (+)<br>NF-κB (−)		
Prevotellaceae (+)	*Prevotella*<br>*Alloprevotella*	*Prevotella_9* (−)	Colitis (−)<br>Inflammation (−)<br>Hypertension (−)	TLR4/MyD88/NF-κB (−)<br>IL-1β (−)<br>IL-6 (−)<br>TNF-α (−)	Acetate (+)<br>Propionate (+)<br>Butyrate (+)	[5,8,15,17,27]
Muribaculaceae (+/-)	*Muribaculum*		Gut barrier function (+)<br>Uric acid excretion (+)<br>Hyperuricemia (−)<br>IBD (−)	ABCG2 (+)<br>GLUT9 (−)<br>SMCT (−)<br>URAT1 (−)<br>IL-10 (+)<br>IL-1β (−)<br>IL-12 (−)<br>IL-18 (−)<br>IL-6 (−)<br>TNF-α (−)	Acetate (+)<br>Propionate (+)<br>Isobutyrate (+)<br>NO (−)	[16,28,34]
Tannerellaceae	*Parabacteroides* (+/−)		Glucose metabolism (+)<br>Lipid metabolism (+)<br>Obesity (−)<br>IBD (−)<br>Gut microenvironment homeostasis (+)	TLR4 (−)<br>IL-10 (+)<br>IL-1β (−)<br>IL-6 (−)<br>TNF-α (−)	SCFAs (+)	[10,25,27,39]
Porphyromonadaceae	*Porphyromonas*		Lipid metabolism (+)<br>Inflammation (−)<br>Colorectal cancer (−)			[40,41]
Odoribacteraceae	*Butyricimonas*		Gut-skin axis homeostasis (+)<br>Skin aging (-)		Butyrate (+)	[17]
**Proteobacteria (Pseudomonadota)**						
Helicobacteraceae (−)	*Helicobacter* (−)	*H. pylori* (−)	Infection (−)<br>Intestinal inflammation (−)<br>IBD (−)<br>Obesity (−)<br>Immunity (+)<br>Gut microenvironment homeostasis (+)<br>oxidative stress (−)<br>Gut barrier function (+)	TLR4/MyD88/NF-κB<br>AMPK/NF-κB<br>IL-1 β (−)<br>IL-6 (−)<br>TNF-α (−)<br>IL-10 (+)<br>MDA (−)<br>CAT (+)<br>T-SOD (+)	Acetate (+)<br>Propionate (+)<br>Butyrate (+)	[8,25,28,30,33]
Desulfovibrionaceae (−)	*Desulfovibrio* (−)		Gut barrier function (+)	TLR4 (−)	Blood metabolites (+)	[3,11,12,25,27,38,42]

Influenced Gut microbiota			Functions & Therapeutics	Involved Proteins/ Cytokine/Pathway	Changes of Metabolites	Reference
Family	Genus	Species				
			Intestinal inflammation (−)<br>Gut microenvironment homeostasis (+)<br>Lipid metabolism (+)<br>Glucose metabolism (+)<br>Insulin sensitivity (+)<br>Reproductive capacity (+)	TNF-α (−)<br>COX-2 (−)<br>IL-1 β (−)<br>IL-6 (−)<br>TNF-α (−)	Acetate (+)<br>Propionate (+)<br>Butyrate (+)	
Enterobacteriaceae (−)	*Escherichia*	*E. coli*	Gut barrier function (+)<br>Mucosal immunity (+)<br>Colitis (−)<br>Infections (−)<br>Inflammation (−)<br>Neuroprotection (+)	TLR4/MyD88/NF-κB (−)<br>AMPK/NF-κB<br>IL-6 (−)<br>TNF-α (−)<br>IFN-γ (−)	LPS (−)<br>SCFA (+)	[7,28,43]
	Salmonella	*S. enteritidis*		IL-1β (−)<br>*S. enteritidis* specific IgA (+)<br>ROS (−)		[44]
	Shigella					[30]
Pseudomonadaceae	*Pseudomonas* (−)	*P. aeruginosa*	Quorum sensing (−)<br>Biofilm formation (−)<br>Infection (−)			[45,46]
Succinivbrionaceae	*Anaerobiospirillum*		Immune homeostasis (+)<br>Inflammation (−)	TLR4 (−)<br>COX-2 (−)<br>TNF-α (−)		[27]
Burkholderiaceae	*Burkholderia*	*B. cenocepacia*	Infection (−)<br>Lipid metabolism (+)<br>Obesity (−)<br>Intestinal inflammation (−)<br>Neuroprotection (+)<br>Gut microenvironment homeostasis (+)<br>Osteoporosis (−)	TLR4 (−)<br>COX-2 (−)<br>TNF-α (−)	SCFAs (+)<br>BAs (+)	[3,13,25,27,37]
	Ralstonia	*R. mannitolilytica*	Infection (−)			[47]
Comamonadaceae	*Comamonas* (−)		Hyperuricemia (−)	ABCG2 (+)<br>GLUT9 (−)<br>URAT1 (−)<br>IL-10 (+)<br>IL-1β (−)<br>IL-12 (−)<br>IL-18 (−)	Acetate (+)<br>Propionate (+)<br>Isobutyrate (+)	[16]
Actinobacteria						
Bifidobacteriaceae	*Bifidobacterium* (+)	*B. gallinarum*	Intestine immunity (+)	IL-6 (−)	SCFAs (+)	[10,13,29,29,37,48]

Influenced Gut microbiota			Functions & Therapeutics	Involved Proteins/ Cytokine/Pathway	Changes of Metabolites	Reference
Family	Genus	Species				
			Gut barrier function (+)<br>Intestinal inflammation (−)<br>Neuroprotection (+)<br>Oxidation (−)<br>Obesity (−)<br>Osteoporosis (−)	IL-1β (−)<br>TNF-α (−)<br>Th17 cells (−)	BAs (+)	
Eggerthellaceae (+)	*Parvibacter* (+)		Inflammation (−)<br>Gut barrier function (+)<br>Infection (−)<br>Glucose metabolism (+)<br>Diabetes (−)	IL-6 (−)<br>IL-1β (−)<br>TNF-α (−)<br>MPO (−)<br>Blood glucose (−)<br>HOMA-IR (−)	Acetate (+)<br>Propionate (+)<br>Butyrate (+)	[11,29]
**Verrucomicrobia**						
Akkermansiaceae	*Akkermansia* (+)	*A. muciniphila*	Immunity (+)<br>Intestinal barrier function (+)<br>Glucose metabolism (+)<br>Lipid metabolism (+)<br>Obesity (−)<br>Inflammation (−)<br>Insulin resistance (−)<br>Oxidation (−)<br>Diabetes (−)<br>Hyperuricemia (−)<br>IBD (−)<br>Metabolic disorders (−)	IL-6 (−)<br>IL-1β (−)<br>TNF-α (−)<br>IFN-γ (−)<br>MDA (−)<br>T-SOD (+)<br>LDL-C (−)<br>TG (−)	Propionate (+)<br>Butyrate (+)	[10,16,48]
Verrucomicrobiaceae			Diabetes (−)			[49]
**Fusobacteria (Fusobacteriota)**						
Fusobacteriaceae	*Cetobacterium (+)*		Intestinal digestion & absorption (+)<br>Glucose metabolism (+)<br>Lipid metabolism (+)	Apoa1 (+)<br>Apoa4 (+)<br>Apoa48 (+)<br>FBP2 (+)<br>DGAT (+)		[50]
	Fusobacterium	*F. nucleatum*	Gut barrier function (+)<br>Inflammation (−)<br>Colorectal cancer (−)	Wnt signaling pathway		[51]

(+) Increasing, upregulating, promoting, accelerating, or maintaining effects of AOS.

(−) Decreasing, downregulating, inhibiting, or suppressing effects of AOS.

References

1. Wang, J.; Zhang, Z.; Dai, T.; Zhang, Z.; Zhang, Q.; Yao, J.; Wang, L.; He, N.; Li, S. The Therapeutic Effect and Possible Mechanisms of Alginate Oligosaccharide on Metabolic Syndrome by Regulating Gut Microbiota. *Food Funct.* **2024**, *15*, 9632–9661, doi:10.1039/D4FO02802C.
2. Wang, X.; Sun, G.; Feng, T.; Zhang, J.; Huang, X.; Wang, T.; Xie, Z.; Chu, X.; Yang, J.; Wang, H.; et al. Sodium Oligomannate Therapeutically Remodels Gut Microbiota and Suppresses Gut Bacterial Amino Acids-Shaped Neuroinflammation to Inhibit Alzheimer's Disease Progression. *Cell Res* **2019**, *29*, 787–803, doi:10.1038/s41422-019-0216-x.
3. Wang, Y.; Li, L.; Ye, C.; Yuan, J.; Qin, S. Alginate Oligosaccharide Improves Lipid Metabolism and Inflammation by Modulating Gut Microbiota in High-Fat Diet Fed Mice. *Appl Microbiol Biotechnol* **2020**, *104*, 3541–3554, doi:10.1007/s00253-020-10449-7.
4. Wu, A.; Gao, Y.; Kan, R.; Ren, P.; Xue, C.; Kong, B.; Tang, Q. Alginate Oligosaccharides Prevent Dextran-Sulfate-Sodium-Induced Ulcerative Colitis via Enhancing Intestinal Barrier Function and Modulating Gut Microbiota. *Foods* **2023**, *12*, 220, doi:10.3390/foods12010220.
5. Zhang, Y.; Deng, X.; Liu, T.; Hu, B.; Yu, B.; Jiang, L.; Wu, Z.; Schroyen, M.; Liu, M. Alginate Oligosaccharides Improve Hepatic Metabolic Disturbance via Regulating the Gut Microbiota. *Food Hydrocolloids* **2024**, *153*, 109980, doi:10.1016/j.foodhyd.2024.109980.
6. Zhang, Y.; Qin, S.; Song, Y.; Yuan, J.; Hu, S.; Chen, M.; Li, L. Alginate Oligosaccharide Alleviated Cisplatin-Induced Kidney Oxidative Stress via Lactobacillus Genus-FAHFAs-Nrf2 Axis in Mice. *Front. Immunol.* **2022**, *13*, 857242, doi:10.3389/fimmu.2022.857242.
7. Zhu La, A.L.T.; Feng, Y.; Hu, D.; Feng, Y.; Jin, X.; Liu, D.; Guo, Y.; Cheng, G.; Hu, Y. Enzymatically Prepared Alginate Oligosaccharides Improve Broiler Chicken Growth Performance by Modulating the Gut Microbiota and Growth Hormone Signals. *J Animal Sci Biotechnol* **2023**, *14*, 96, doi:10.1186/s40104-023-00887-4.
8. Shen, Z.; Yin, H.; Sun, L.; Chen, L.; Li, J.; Zhang, X.; Zeng, M.; Jiang, X.; Yu, J. Influence of Consumption of Unsaturated Alginate Oligosaccharides on the Gut Microbiota and the Intestinal Mucosal Immunity Homeostasis in Immunocompromised Mice. *Carbohydrate Polymer Technologies and Applications* **2024**, *8*, 100604, doi:10.1016/j.carpta.2024.100604.
9. Han, H.; Zhou, Y.; Xiong, B.; Zhong, R.; Jiang, Y.; Sun, H.; Tan, J.; Zhang, B.; Guan, C.; Schroyen, M.; et al. Alginate Oligosaccharides Increase Boar Semen Quality by Affecting Gut Microbiota and Metabolites in Blood and Sperm. *Front. Microbiol.* **2022**, *13*, 982152, doi:10.3389/fmicb.2022.982152.
10. Li, S.; Wang, L.; Liu, B.; He, N. Unsaturated Alginate Oligosaccharides Attenuated Obesity-Related Metabolic Abnormalities by Modulating Gut Microbiota in High-Fat-Diet Mice. *Food Funct.* **2020**, *11*, 4773–4784, doi:10.1039/C9FO02857A.
11. Qiang, T.; Wang, J.; Jiang, L.; Xiong, K. Modulation of Hyperglycemia by Sodium Alginate Is Associated with Changes of Serum Metabolite and Gut Microbiota in Mice. *Carbohydrate Polymers* **2022**, *291*, 119359, doi:10.1016/j.carbpol.2022.119359.
12. Zhang, P.; Liu, J.; Xiong, B.; Zhang, C.; Kang, B.; Gao, Y.; Li, Z.; Ge, W.; Cheng, S.; Hao, Y.; et al. Microbiota from Alginate Oligosaccharide-Dosed Mice Successfully Mitigated Small Intestinal Mucositis. *Microbiome* **2020**, *8*, 112, doi:10.1186/s40168-020-00886-x.

13. Bosch, M.E.; Dodiya, H.B.; Michalkiewicz, J.; Lee, C.; Shaik, S.M.; Weigle, I.Q.; Zhang, C.; Osborn, J.; Nambiar, A.; Patel, P.; et al. Sodium Oligomannate Alters Gut Microbiota, Reduces Cerebral Amyloidosis and Reactive Microglia in a Sex-Specific Manner. *Mol Neurodegeneration* **2024**, *19*, 18, doi:10.1186/s13024-023-00700-w.
14. Chen, X.; Chen, X.; Yan, D.; Zhang, N.; Fu, W.; Wu, M.; Ge, F.; Wang, J.; Li, X.; Geng, M.; et al. GV-971 Prevents Severe Acute Pancreatitis by Remodeling the Microbiota-Metabolic-Immune Axis. *Nat Commun* **2024**, *15*, 8278, doi:10.1038/s41467-024-52398-z.
15. Han, Z.-L.; Chen, M.; Fu, X.-D.; Yang, M.; Hrmova, M.; Zhao, Y.-H.; Mou, H.-J. Potassium Alginate Oligosaccharides Alter Gut Microbiota, and Have Potential to Prevent the Development of Hypertension and Heart Failure in Spontaneously Hypertensive Rats. *IJMS* **2021**, *22*, 9823, doi:10.3390/ijms22189823.
16. Wei, B.; Ren, P.; Yang, R.; Gao, Y.; Tang, Q.; Xue, C.; Wang, Y. Ameliorative Effect of Mannuronate Oligosaccharides on Hyperuricemic Mice via Promoting Uric Acid Excretion and Modulating Gut Microbiota. *Nutrients* **2023**, *15*, 417, doi:10.3390/nu15020417.
17. Gao, T.; Li, Y.; Wang, X.; Ren, F. Alginate Oligosaccharide-Mediated Butyrate-HIF-1α Axis Improves Skin Aging in Mice. *Journal of Pharmaceutical Analysis* **2024**, *14*, 100911, doi:10.1016/j.jpha.2023.12.001.
18. Cenit, M.C.; Sanz, Y.; Codoñer-Franch, P. Influence of Gut Microbiota on Neuropsychiatric Disorders. *WJG* **2017**, *23*, 5486, doi:10.3748/wjg.v23.i30.5486.
19. Czepiel, J.; Dróżdż, M.; Pituch, H.; Kuijper, E.J.; Perucki, W.; Mielimonka, A.; Goldman, S.; Wultańska, D.; Garlicki, A.; Biesiada, G. Clostridium Difficile Infection: Review. *Eur J Clin Microbiol Infect Dis* **2019**, *38*, 1211–1221, doi:10.1007/s10096-019-03539-6.
20. Gagnière, J. Gut Microbiota Imbalance and Colorectal Cancer. *WJG* **2016**, *22*, 501, doi:10.3748/wjg.v22.i2.501.
21. Raskov, H.; Burcharth, J.; Pommergaard, H.-C. Linking Gut Microbiota to Colorectal Cancer. *J. Cancer* **2017**, *8*, 3378–3395, doi:10.7150/jca.20497.
22. Vaiserman, A.M.; Koliada, A.K.; Marotta, F. Gut Microbiota: A Player in Aging and a Target for Anti-Aging Intervention. *Ageing Research Reviews* **2017**, *35*, 36–45, doi:10.1016/j.arr.2017.01.001.
23. Yang, W.-Y.; Lee, Y.; Lu, H.; Chou, C.-H.; Wang, C. Analysis of Gut Microbiota and the Effect of Lauric Acid against Necrotic Enteritis in Clostridium Perfringens and Eimeria Side-by-Side Challenge Model. *PLoS ONE* **2019**, *14*, e0205784, doi:10.1371/journal.pone.0205784.
24. Wang, J.; Liao, Y.; Qin, J.; Ma, C.; Jin, Y.; Wang, X.; Chen, K.; Ouyang, P. Increasing Lysine Level Improved Methanol Assimilation toward Butyric Acid Production in Butyribacterium Methylotrophicum. *Biotechnol Biofuels* **2023**, *16*, 10, doi:10.1186/s13068-023-02263-w.
25. Yan, S.; Zhu, Y.; Li, L.; Qin, S.; Yuan, J.; Chang, X.; Hu, S. Alginate Oligosaccharide Ameliorates Azithromycin-Induced Gut Microbiota Disorder *via Bacteroides Acidifaciens* -FAHFAs and *Bacteroides* -TCA Cycle Axes. *Food Funct.* **2023**, *14*, 427–444, doi:10.1039/D2FO02812C.
26. Lu, S.; Tao, Z.; Wang, G.; Na, K.; Wu, L.; Zhang, L.; Li, X.; Guo, X. Mannuronate Oligosaccharides Ameliorate Experimental Colitis and Secondary Neurological Dysfunction by Manipulating the Gut–Brain Axis. *J. Agric. Food Chem.* **2025**, *73*, 2935–2950, doi:10.1021/acs.jafc.4c10378.
27. He, N.; Yang, Y.; Wang, H.; Liu, N.; Yang, Z.; Li, S. Unsaturated Alginate Oligosaccharides (UAOS) Protects against Dextran Sulfate Sodium-Induced Colitis Associated with Regulation of Gut Microbiota. *Journal of Functional Foods* **2021**, *83*, 104536, doi:10.1016/j.jff.2021.104536.

28. Zhang, Y.; Guo, C.; Li, Y.; Han, X.; Luo, X.; Chen, L.; Zhang, T.; Wang, N.; Wang, W. Alginate Oligosaccharides Ameliorate DSS-Induced Colitis through Modulation of AMPK/NF-κB Pathway and Intestinal Microbiota. *Nutrients* **2022**, *14*, 2864, doi:10.3390/nu14142864.
29. Mi, J.; Tong, Y.; Zhang, Q.; Wang, Q.; Wang, Y.; Wang, Y.; Lin, G.; Ma, Q.; Li, T.; Huang, S. Alginate Oligosaccharides Enhance Gut Microbiota and Intestinal Barrier Function, Alleviating Host Damage Induced by Deoxynivalenol in Mice. *The Journal of Nutrition* **2024**, *154*, 3190–3202, doi:10.1016/j.tjnut.2024.09.031.
30. Li, J.; Shao, M.; Liu, H.; Guo, P.; Liu, F.; Ma, M.; Li, Q. Lithium Coupled with C6-Carboxyl Improves the Efficacy of Oligoguluronate in DSS-Induced Ulcerative Colitis in C57BL/6J Mice. *Marine Drugs* **2024**, *22*, 573, doi:10.3390/md22120573.
31. Zhao, L.; Zhang, X.; Zhou, Y.; Fu, K.; Lau, H.C.-H.; Chun, T.W.-Y.; Cheung, A.H.-K.; Coker, O.O.; Wei, H.; Wu, W.K.-K.; et al. Parvimonas Micra Promotes Colorectal Tumorigenesis and Is Associated with Prognosis of Colorectal Cancer Patients. *Oncogene* **2022**, *41*, 4200–4210, doi:10.1038/s41388-022-02395-7.
32. Li, J.; Guo, Y.; Ma, L.; Liu, Y.; Zou, C.; Kuang, H.; Han, B.; Xiao, Y.; Wang, Y. Synergistic Effects of Alginate Oligosaccharide and Cyanidin-3-O-Glucoside on the Amelioration of Intestinal Barrier Function in Mice. *Food Science and Human Wellness* **2023**, *12*, 2276–2285, doi:10.1016/j.fshw.2023.03.047.
33. Liu, M.; Deng, X.; Zhao, Y.; Everaert, N.; Zhang, H.; Xia, B.; Schroyen, M. Alginate Oligosaccharides Enhance Antioxidant Status and Intestinal Health by Modulating the Gut Microbiota in Weaned Piglets. *IJMS* **2024**, *25*, 8029, doi:10.3390/ijms25158029.
34. Wei, B.; Ren, P.; Xue, C.; Wang, Y.; Tang, Q. Guluronate Oligosaccharides Exerts Beneficial Effects on Hyperuricemia and Regulation of Gut Microbiota in Mice. *Food Bioscience* **2023**, *54*, 102855, doi:10.1016/j.fbio.2023.102855.
35. Zhou, Y.; Wei, Z.; Tan, J.; Sun, H.; Jiang, H.; Gao, Y.; Zhang, H.; Schroyen, M. Alginate Oligosaccharide Extends the Service Lifespan by Improving the Sperm Metabolome and Gut Microbiota in an Aging Duroc Boars Model. *Front. Cell. Infect. Microbiol.* **2023**, *13*, 1308484, doi:10.3389/fcimb.2023.1308484.
36. Mushtaq, N.; Hussain, S.; Zhang, S.; Yuan, L.; Li, H.; Ullah, S.; Wang, Y.; Xu, J. Molecular Characterization of Alterations in the Intestinal Microbiota of Patients with Grade 3 Hypertension. *Int J Mol Med* **2019**, doi:10.3892/ijmm.2019.4235.
37. Zhang, M.; Sun, J.; Zhao, H.; Liu, Y.; Tang, Z.; Wen, Y.; Ma, Q.; Zhang, L.; Zhang, Y. Alginate Oligosaccharides Relieve Estrogen-Deprived Osteosarcopenia by Affecting Intestinal Th17 Differentiation and Systemic Inflammation through the Manipulation of Bile Acid Metabolism. *International Journal of Biological Macromolecules* **2025**, *295*, 139581, doi:10.1016/j.ijbiomac.2025.139581.
38. Zheng, W.; Duan, M.; Jia, J.; Song, S.; Ai, C. Low-Molecular Alginate Improved Diet-Induced Obesity and Metabolic Syndrome through Modulating the Gut Microbiota in BALB/c Mice. *International Journal of Biological Macromolecules* **2021**, *187*, 811–820, doi:10.1016/j.ijbiomac.2021.08.003.
39. Lu, S.; Na, K.; Wei, J.; Tao, T.; Zhang, L.; Fang, Y.; Li, X.; Guo, X. Alginate Oligosaccharide Structures Differentially Affect DSS-Induced Colitis in Mice by Modulating Gut Microbiota. *Carbohydrate Polymers* **2023**, *312*, 120806, doi:10.1016/j.carbpol.2023.120806.
40. Tavella, T.; Rampelli, S.; Guidarelli, G.; Bazzocchi, A.; Gasperini, C.; Pujos-Guillot, E.; Comte, B.; Barone, M.; Biagi, E.; Candela, M.; et al. Elevated Gut Microbiome Abundance of *Christensenellaceae, Porphyromonadaceae and Rikenellaceae* Is Associated with Reduced Visceral Adipose Tissue and Healthier Metabolic Profile in Italian Elderly. *Gut Microbes* **2021**, *13*, 1880221, doi:10.1080/19490976.2021.1880221.

41. Wong, C.C.; Yu, J. Gut Microbiota in Colorectal Cancer Development and Therapy. *Nature Reviews Clinical Oncology* **2023**, *20*, 429–452, doi:10.1038/s41571-023-00766-x.
42. Zhao, Y.; Zhang, P.; Ge, W.; Feng, Y.; Li, L.; Sun, Z.; Zhang, H.; Shen, W. Alginate Oligosaccharides Improve Germ Cell Development and Testicular Microenvironment to Rescue Busulfan Disrupted Spermatogenesis. *Theranostics* **2020**, *10*, 3308–3324, doi:10.7150/thno.43189.
43. Wan, J.; Zhang, J.; Xu, Q.; Yin, H.; Chen, D.; Yu, B.; He, J. Alginate Oligosaccharide Protects against Enterotoxigenic Escherichia Coli-Induced Porcine Intestinal Barrier Injury. *Carbohydrate Polymers* **2021**, *270*, 118316, doi:10.1016/j.carbpol.2021.118316.
44. Yan, G.L.; Guo, Y.M.; Yuan, J.M.; Liu, D.; Zhang, B.K. Sodium Alginate Oligosaccharides from Brown Algae Inhibit Salmonella Enteritidis Colonization in Broiler Chickens. *Poultry Science* **2011**, *90*, 1441–1448, doi:10.3382/ps.2011-01364.
45. Hengzhuang, W.; Song, Z.; Ciofu, O.; Onsøyen, E.; Rye, P.D.; Høiby, N. OligoG CF-5/20 Disruption of Mucoid Pseudomonas Aeruginosa Biofilm in a Murine Lung Infection Model. *Antimicrob Agents Chemother* **2016**, *60*, 2620–2626, doi:10.1128/AAC.01721-15.
46. Jack, A.A.; Khan, S.; Powell, L.C.; Pritchard, M.F.; Beck, K.; Sadh, H.; Sutton, L.; Cavaliere, A.; Florance, H.; Rye, P.D.; et al. Alginate Oligosaccharide-Induced Modification of the *lasI-lasR* and *rhlI-rhlR* Quorum-Sensing Systems in Pseudomonas Aeruginosa. *Antimicrob Agents Chemother* **2018**, *62*, e02318-17, doi:10.1128/AAC.02318-17.
47. Green, H.D.; Bright-Thomas, R.; Kenna, D.T.; Turton, J.F.; Woodford, N.; Jones, A.M. Ralstonia Infection in Cystic Fibrosis. *Epidemiol. Infect.* **2017**, *145*, 2864–2872, doi:10.1017/S0950268817001728.
48. Li, T.; Huang, S.; Wang, J.; Yin, P.; Liu, H.; Sun, C. Alginate Oligosaccharides Protect against Fumonisin B1-Induced Intestinal Damage via Promoting Gut Microbiota Homeostasis. *Food Research International* **2022**, *152*, 110927, doi:10.1016/j.foodres.2021.110927.
49. Peng, W.; Huang, J.; Yang, J.; Zhang, Z.; Yu, R.; Fayyaz, S.; Zhang, S.; Qin, Y. Integrated 16S rRNA Sequencing, Metagenomics, and Metabolomics to Characterize Gut Microbial Composition, Function, and Fecal Metabolic Phenotype in Non-Obese Type 2 Diabetic Goto-Kakizaki Rats. *Front. Microbiol.* **2020**, *10*, 3141, doi:10.3389/fmicb.2019.03141.
50. Li, F.; Tang, Y.; Wei, L.; Yang, M.; Lu, Z.; Shi, F.; Zhan, F.; Li, Y.; Liao, W.; Lin, L.; et al. Alginate Oligosaccharide Modulates Immune Response, Fat Metabolism, and the Gut Bacterial Community in Grass Carp (Ctenopharyngodon Idellus). *Fish & Shellfish Immunology* **2022**, *130*, 103–113, doi:10.1016/j.fsi.2022.08.067.
51. Rubinstein, M.R.; Wang, X.; Liu, W.; Hao, Y.; Cai, G.; Han, Y.W. Fusobacterium Nucleatum Promotes Colorectal Carcinogenesis by Modulating E-Cadherin/β-Catenin Signaling via Its FadA Adhesin. *Cell Host & Microbe* **2013**, *14*, 195–206, doi:10.1016/j.chom.2013.07.012.
